# Inhibition of antiapoptotic BCL-2 proteins with ABT-263 induces fibroblast apoptosis, reversing persistent pulmonary fibrosis

**DOI:** 10.1172/jci.insight.163762

**Published:** 2023-02-08

**Authors:** Joseph C. Cooley, Nomin Javkhlan, Jasmine A. Wilson, Daniel G. Foster, Benjamin L. Edelman, Luis A. Ortiz, David A. Schwartz, David W.H. Riches, Elizabeth F. Redente

**Affiliations:** 1 Division of Pulmonary, Critical Care and Sleep Medicine, Department of Medicine, National Jewish Health, Denver, Colorado, USA.; 2 Division of Pulmonary Sciences and Critical Care Medicine, Department of Medicine, University of Colorado Anschutz Medical Campus, Aurora, Colorado, USA.; 3 Program in Cell Biology, Department of Pediatrics, National Jewish Health, Denver, Colorado, USA.; 4 Department of Pharmaceutical Sciences, University of Colorado Anschutz Medical Campus, Aurora, Colorado, USA.; 5 Department of Environmental and Occupational Health, Graduate School of Public Health at the University of Pittsburgh, Pittsburgh, Pennsylvania, USA.; 6 Department of Immunology and Microbiology, University of Colorado Anschutz Medical Campus, Aurora, Colorado, USA.; 7 Department of Research, Veterans Affairs Eastern Colorado Health Care System, Aurora, Colorado, USA.

**Keywords:** Cell Biology, Pulmonology, Apoptosis pathways, Fibrosis, Mouse models

## Abstract

Patients with progressive fibrosing interstitial lung diseases (PF-ILDs) carry a poor prognosis and have limited therapeutic options. A hallmark feature is fibroblast resistance to apoptosis, leading to their persistence, accumulation, and excessive deposition of extracellular matrix. A complex balance of the B cell lymphoma 2 (BCL-2) protein family controlling the intrinsic pathway of apoptosis and fibroblast reliance on antiapoptotic proteins has been hypothesized to contribute to this resistant phenotype. Examination of lung tissue from patients with PF-ILD (idiopathic pulmonary fibrosis and silicosis) and mice with PF-ILD (repetitive bleomycin and silicosis) showed increased expression of antiapoptotic BCL-2 family members in α–smooth muscle actin–positive fibroblasts, suggesting that fibroblasts from fibrotic lungs may exhibit increased susceptibility to inhibition of antiapoptotic BCL-2 family members BCL-2, BCL-XL, and BCL-W with the BH3 mimetic ABT-263. We used 2 murine models of PF-ILD to test the efficacy of ABT-263 in reversing established persistent pulmonary fibrosis. Treatment with ABT-263 induced fibroblast apoptosis, decreased fibroblast numbers, and reduced lung collagen levels, radiographic disease, and histologically evident fibrosis. Our studies provide insight into how fibroblasts gain resistance to apoptosis and become sensitive to the therapeutic inhibition of antiapoptotic proteins. By targeting profibrotic fibroblasts, ABT-263 offers a promising therapeutic option for PF-ILDs.

## Graphical abstract

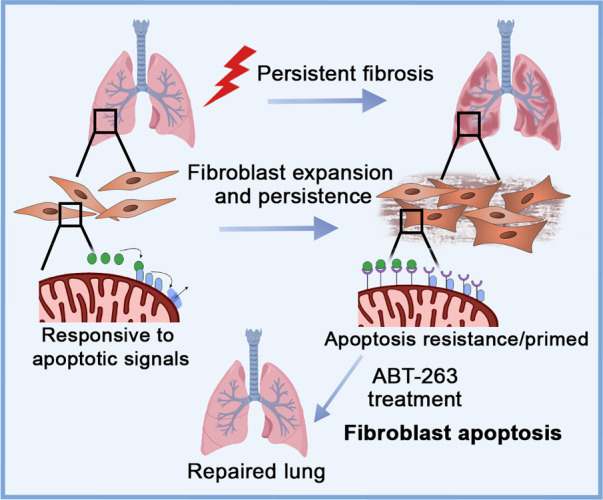

## Introduction

Pulmonary fibrosis is the final common pathologic outcome of multiple insults and diseases. A subset of patients with pulmonary fibrosis develop a progressive phenotype, known as progressive fibrosing interstitial lung disease (PF-ILD). The PF-ILD phenotype can be seen with idiopathic pulmonary fibrosis (IPF) and pneumonoconiosis including silicosis, connective tissue disease, hypersensitivity pneumonitis, sarcoidosis, and nonspecific interstitial pneumonia (1). Regardless of etiology, PF-ILDs share certain pathophysiologic and clinical characteristics, including progressive decline in lung function, reduced quality of life, and early mortality (1). There are few therapeutic options and none have a clear mortality benefit (2, 3). Therefore, there remains an urgent need to understand the fundamental mechanisms of fibrosis progression and to develop targeted therapeutic options for these debilitating diseases.

In normal wound healing, α–smooth muscle actin–positive (α-SMA^+^) myofibroblasts produce, organize, and remodel the extracellular matrix (ECM), forming a physiologic scar (4). Once wound repair is complete, myofibroblasts are cleared via apoptosis or dedifferentiation (4). However, in pathologic fibrosis, myofibroblasts become resistant to apoptotic signals, resulting in their persistence and the continuation of scar deposition (5–8). In vitro studies by ourselves and others have shown that fibrotic lung fibroblasts isolated from patients with IPF are resistant to apoptosis through decreased expression of the death receptor Fas and concurrent increased expression of antiapoptotic genes, including B cell lymphoma 2 (*BCL-2*), *PTPN13*, and *XIAP* (6, 9, 10), which together impair apoptotic signaling (6–8, 11–13). In vivo, we have recently demonstrated that loss of Fas in fibroblasts is sufficient to prevent extrinsic apoptosis and results in impaired homeostatic fibrosis resolution after a single instillation of bleomycin (12). Together, these studies have established that fibroblast acquisition of an apoptosis-resistant phenotype promotes accumulation in fibrotic lungs. However, the effects of inhibiting the intrinsic apoptosis pathway in fibroblasts on fibrosis resolution in established persistent, progressive pulmonary fibrosis remains a key gap in knowledge.

The intrinsic pathway of apoptosis is controlled by the BCL-2 family of proteins made up of antiapoptotic and proapoptotic members (activators, sensitizers, and pore formers) (14). While altered regulation of the BCL-2 family members is a well-known mechanism for oncogenesis (15), the role of these complex interactions in fibroblast persistence has not been thoroughly studied in human fibrotic lung disease or animal models of progressive pulmonary fibrosis. In IPF, increased levels of the antiapoptotic protein BCL-2 have been associated with fibroblast resistance to apoptosis in fibrotic lung disease (9, 16). Additionally, an increased ratio of BCL-2 to the proapoptotic protein BCL2-associated X (BAX) has been measured in fibroblasts isolated from patients with nonresolving fibroproliferative acute respiratory distress syndrome (ARDS) compared with resolving ARDS (17). Convergence of the intrinsic and extrinsic apoptotic pathways can occur when Fas-mediated activation of caspase-8 cleaves BH3 interacting domain death agonist (BID) into tBID (a BH3 activator), leading to the release of cytochrome *c* from the mitochondria, highlighting the complexity of crosstalk between these pathways (18–20). Additionally, XIAP can regulate both intrinsic and extrinsic apoptosis. It has been shown to function as an E3 ligase for BCL-2, promoting its ubiquitination and degradation, thus promoting apoptosis and furthering the complex crosstalk that may regulate fibroblast resistance to apoptosis (21, 22).

As cells become resistant to apoptotic signals through increased antiapoptotic BCL-2 family member expression, which act by sequestering proapoptotic family members, they enter a state of survival close to the apoptotic threshold, defined as “primed” (15, 23). If the proapoptotic proteins are released, for example through inhibition of the antiapoptotic BCL-2 family members, they tip the cell over the apoptotic threshold, thus inducing cell death (15, 23). Priming is important because it not only describes the proximity of a cell to the apoptotic threshold but also predicts sensitivity to BH3 mimetics that target the antiapoptotic BCL-2 family proteins (24). Each BCL-2 family protein contains 1 or more BCL-2 homology domains (BH1, BH2, BH3, BH4) (24). ABT-263 (navitoclax) is a BH3 mimetic drug that inhibits the antiapoptotic proteins BCL-2, BCL-XL, and BCL-W (23) and has been studied in several clinical trials as treatment for malignant disorders (25). We hypothesized that in progressive pulmonary fibrosis, fibroblasts become increasingly primed, leaving them sensitive to ABT-263–induced apoptosis.

ABT-263 has recently been shown to attenuate the development of pulmonary fibrosis after single-dose bleomycin in mice (26) and to reduce dermal fibrosis in a model of bleomycin-induced scleroderma (27). A single dose of bleomycin is the most frequently used mouse model of pulmonary fibrosis. However, peak fibroblast apoptosis occurs at approximately 3–4 weeks, with fibroblast numbers normalizing and fibrosis resolving by approximately 9 weeks (12, 28), making it a model of homeostatic fibrosis resolution as it fails to develop the persistent, progressive fibrosis seen in PF-ILD. To effectively study the role of fibroblast resistance to apoptosis in persistent, progressive fibrosis, the model used must recapitulate fundamental aspects of human disease. Therefore, we chose to use repetitive bleomycin (29) and silicosis (30) as mouse models of persistent, progressive fibrosis.

Herein, we demonstrate that antiapoptotic BCL-2 family members are expressed by fibroblasts in both human and murine progressive fibrotic lung disease, that these fibroblasts are highly primed, and that treatment with ABT-263 induced fibroblast apoptosis and reduced fibrosis in 2 preclinical models of PF-ILD.

## Results

### Antiapoptotic BCL-2 family members are expressed by α-SMA^+^ fibrotic fibroblasts in IPF, silicosis, and murine models of PF-ILD.

**Figure 1:**
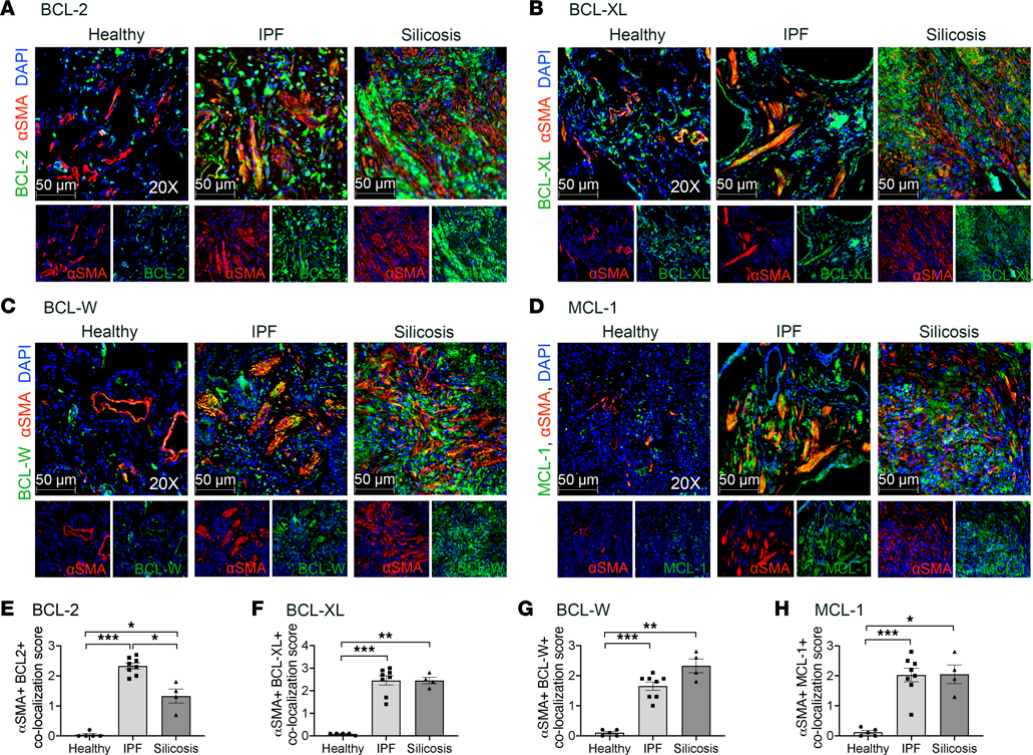
α-SMA^+^ fibrotic fibroblasts express antiapoptotic BCL-2 family members in IPF and silicosis. Immunofluorescence imaging of lungs from healthy donors, IPF, and silicosis for anti–α-SMA (red), DAPI (blue), and antiapoptotic BCL-2 family members (green): (**A**) BCL-2, (**B**) BCL-XL, (**C**) BCL-W, (**D**) MCL-1. (**E**–**H**) Semiquantitative scoring of colocalization of antiapoptotic BCL-2 family members in α-SMA^+^ cells: 0 (0%), 1 (1%–33%), 2 (34%–66%), 3 (67%–100%). *n* = 4–8 individuals per group. Ten images per slide were scored. Graphed as scatterplot with bar, mean ± SEM. *P < 0.05, **P < 0.01, ***P < 0.001, Brown-Forsythe and Welch’s ANOVA with Dunnett’s correction for multiple comparisons. Total magnification with objective 200×.

α-SMA^+^ fibrotic fibroblasts are key profibrotic effector cells in pathologic scarring (4). Therefore, we sought to determine if α-SMA^+^ fibrotic fibroblasts express antiapoptotic BCL-2 family members in human PF-ILDs, IPF, and silicosis (Supplemental Table 1; supplemental material available online with this article; https://doi.org/10.1172/jci.insight.163762DS1), by co-immunostaining human tissue from healthy donors, IPF, and silicosis for α-SMA and antiapoptotic BCL-2 family members. Figure 1 and Supplemental Figure 1 show that in IPF and silicosis, α-SMA^+^ cells expressed BCL-2, BCL-XL, BCL-W, and MCL-1 at a higher frequency compared with α-SMA^+^ cells in healthy lungs. Compared with silicosis lungs, α-SMA^+^ cells in IPF lungs expressed BCL-2 more frequently (Figure 1E). Co-immunostaining for α-SMA^+^ cells and the pore formers BAX and BCL-2 antagonist/killer (BAK) n healthy and IPF lungs did not demonstrate significant colocalization (Supplemental Figure 2). To correlate our immunostaining data with primary lung fibroblasts isolated from healthy controls and patients with IPF and cultured in vitro, we examined a published RNA-sequencing data set (GSE44723) (31). Samples were categorized as healthy controls, IPF with slow progression of disease, IPF with rapid progression, and all IPF combined. Between healthy and IPF groups there were increases in *BCL-2*, *BCL-XL*, and proapoptotic member *BAK* (Supplemental Figure 3).

**Figure 2:**
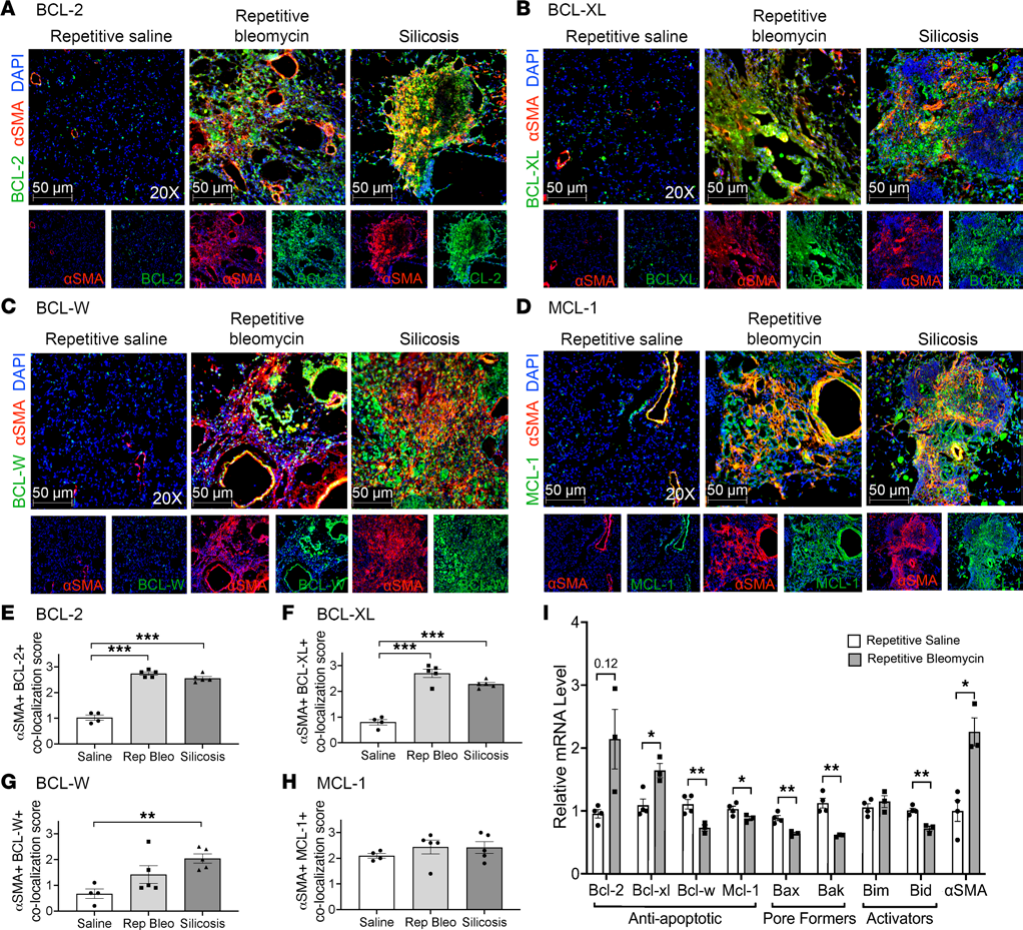
In 2 preclinical models of PF-ILD, α-SMA^+^ fibrotic fibroblasts express antiapoptotic BCL-2 family members. Immunofluorescence imaging of murine lungs from repetitive saline, repetitive bleomycin (28-week), and silicosis (24-week) mice for anti-α-SMA (red), DAPI (blue), and antiapoptotic BCL-2 family members (green): (**A**) BCL-2, (**B**) BCL-XL, (**C**) BCL-W, (**D**) MCL-1. (**E**–**H**) Semiquantitative scoring of colocalization of antiapoptotic BCL-2 family members in α-SMA^+^ cells: 0 (0%), 1 (1%–33%), 2 (34%–66%), 3 (67%–100%). *n* = 4–5 mice per group. A total of 10 images per slide were scored. (**I**) Quantitative PCR (qPCR) of BCL-2 family members and α-SMA in ex vivo–sorted primary lung fibroblasts from mice (28-week repetitive saline or 28-week repetitive bleomycin). *n* = 3–4 mice per group. Graphed as scatterplot with bar, mean ± SEM. *P < 0.05, **P < 0.01, ***P < 0.001, Brown-Forsythe and Welch’s ANOVA with Dunnett’s correction for multiple comparisons or 2-tailed *t* test with Welch’s correction. Total magnification with objective 200×.

Next, we assessed α-SMA^+^ fibrotic fibroblast expression of antiapoptotic BCL-2 family members in 2 murine models of PF-ILD, repetitive bleomycin and silicosis. Similar to α-SMA^+^ fibrotic fibroblasts in human PF-ILD, murine α-SMA^+^ cells had significantly more colocalization with BCL-2, BCL-XL, and BCL-W during established and progressive disease 24–28 weeks after fibrosis initiation (Figure 2 and Supplemental Figure 4) compared with age-matched saline controls and very limited colocalization for BAX or BAK (Supplemental Figure 2). The same expression pattern of antiapoptotic members was seen early in disease with 10-week repetitive bleomycin and 4-week silica mice (Supplemental Figure 5), suggesting an early switch to an apoptosis-resistant phenotype. To determine the relative gene expression of antiapoptotic and proapoptotic BCL-2 family members, lineage-negative (Lin^–^) pulmonary fibroblasts were isolated from mice instilled with repetitive saline or repetitive bleomycin after 28 weeks and assessed ex vivo by qPCR for *Bcl-2* family members without expansion on tissue culture plastic. We found significant differences in both the pro- and antiapoptotic members of the *Bcl-2* family (Figure 2I). Fibrotic fibroblasts had a significant increase in the antiapoptotic gene *Bcl-xl* and a trend toward increased *Bcl-2* (*P* = 0.12), with a decrease in the antiapoptotic genes *Bcl-w* and *Mcl-1* compared with naive fibroblasts. Fibrotic fibroblasts also had significantly decreased levels of the proapoptotic genes *Bid* (activator), *Bax*, and *Bak* (pore formers). Taken together, in fibrotic lung disease, fibrotic fibroblasts have a substantially altered BCL-2 family member profile, both by gene and protein expression compared with normal fibroblasts, supporting a phenotype that is resistant to apoptosis.

### Fibrotic fibroblasts are more susceptible to ABT-263, likely due to alterations in BCL-2 family members.

**Figure 3:**
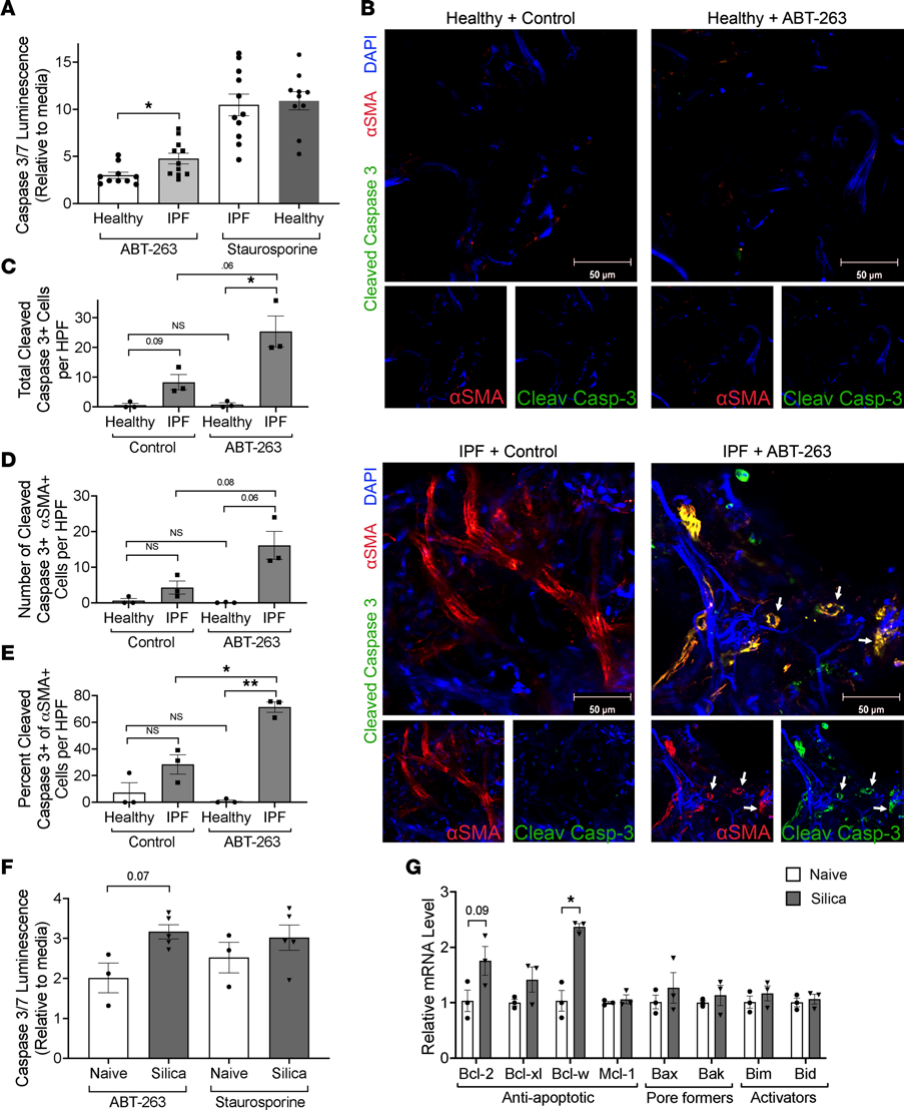
In vitro and ex vivo inhibition of antiapoptotic BCL-2 family members causes fibroblast apoptosis and to a greater degree in fibrotic fibroblasts. (**A**) Primary lung fibroblasts from healthy and IPF donors were treated with ABT-263, and apoptosis was measured by caspase-3/7 activity. *n* = 3 individuals, 3–4 experimental replicates per individual. (**B**) PCLS from fresh healthy and IPF lungs were treated with ABT-263 and stained for anti–cleaved caspase-3 (green), α-SMA (red), and DAPI (blue) and counted for (**C**) total cleaved caspase-3–positive cells, (**D**) total α-SMA cleaved caspase-3 double-positive cells, and (**E**) the percentage of α-SMA^+^ cells that were also cleaved caspase-3 positive. *n* = 3 individuals, 5 images per sample were counted. White arrows indicate α-SMA/cleaved caspase-3 double-positive cells. (**F**) Primary lung fibroblasts from naive and 8-week silica mice were exposed to ABT-263, and apoptosis was measured by caspase-3/7 activity. *n* = 3–5. (**G**) qPCR results from in vitro–cultured fibroblasts from naive and 8-week silica mice. *n* = 3 per group. Graphed as scatterplot with bar, mean ± SEM. *P < 0.05, **P < 0.01, Brown-Forsythe and Welch’s ANOVA with Dunnett’s correction for multiple comparisons or 2-tailed *t* test with Welch’s correction.

Next, we evaluated if ABT-263 would induce a greater apoptotic response in primary lung fibroblasts isolated from fibrotic lungs compared with healthy lungs. IPF-derived primary lung fibroblasts had a 1.6-fold increase in caspase-3/7 activity in response to treatment with ABT-263 (1 μM) compared with healthy fibroblasts (Figure 3A). Because in vitro culture conditions can alter BCL-2 family member expression, including high substrate stiffness like that of plastic (27), we sought to examine the effects of ABT-263 on fibroblasts in precision-cut lung slices (PCLS) from freshly explanted healthy and IPF lung tissue. PCLS were treated with ABT-263 and immunostained for α-SMA and cleaved caspase-3 to identify fibroblasts undergoing apoptosis (Figure 3, B–E, and Supplemental Table 2). Compared with control solution, ABT-263 induced greater apoptosis of α-SMA^+^ fibroblasts in IPF PCLS. Healthy PCLS had minimal apoptotic cells after treatment with ABT-263. Additionally, there was a trend toward increased baseline apoptosis between control-treated healthy PCLS and IPF PCLS.

Similarly, we evaluated the apoptosis effect of ABT-263 (5 μM) on primary murine lung fibrotic fibroblasts isolated 8 weeks after silica and found that treatment resulted in a 1.58-fold increase in caspase-3/7 activity compared with naive fibroblasts (*P* = 0.07) (Figure 3F). We were unable to perform this assay on fibrotic primary lung fibroblasts isolated 28 weeks after repetitive bleomycin, as they were not able to be expanded after passage 3 and had increased expression of senescence-associated galactosidase compared with naive fibroblasts, suggesting a senescent phenotype (Supplemental Figure 6). To determine why fibrotic murine fibrotic fibroblasts were more susceptible to apoptosis after treatment with ABT-263, we quantified BCL-2 family member expression by qPCR analysis on in vitro–cultured fibroblasts from naive and fibrotic mice. Compared with naive lungs, fibroblasts from silica-exposed lungs had significantly increased expression of *Bcl-w* (*P* < 0.05), with a trend toward a significant increase in *Bcl-2* (*P* = 0.09) (Figure 3G). Taken together, fibroblasts isolated from fibrotic lungs, from both humans and mice, had increased gene expression of the antiapoptotic BCL-2 family members compared with healthy lung fibroblasts. The observed increases in gene and protein expression are the same antiapoptotic BCL-2 family members targeted by ABT-263, providing rationale for the increased sensitivity of in vitro human and mouse fibrotic fibroblasts, and IPF-associated fibroblasts, in PCLS to ABT-263.

### Fibrotic fibroblasts from mice with persistent, progressive fibrosis are more primed than fibroblasts from naive mice.

**Figure 4:**
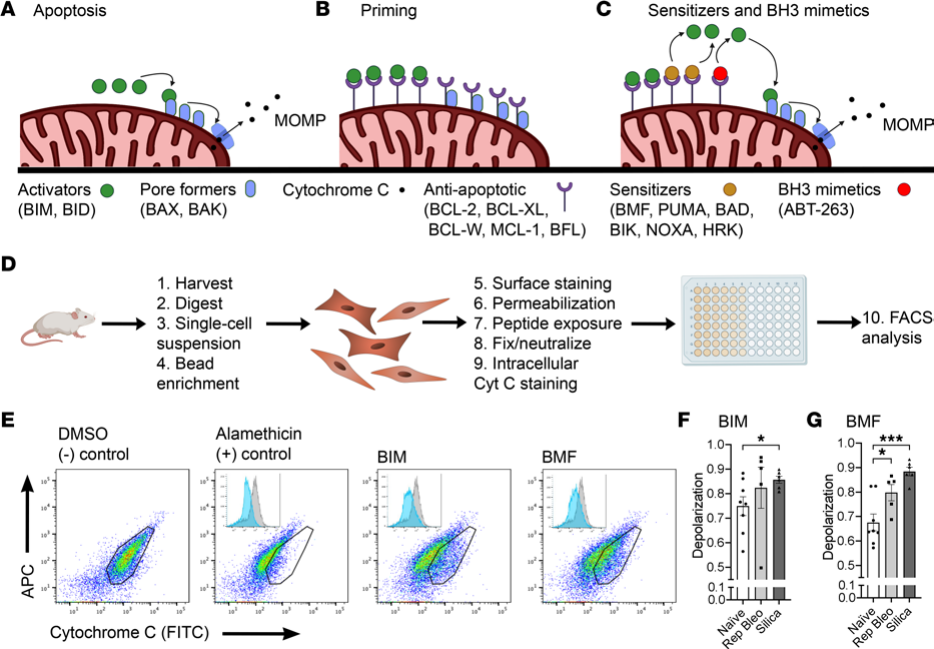
Ex vivo fibrotic fibroblasts are more primed than those from naive mice. (**A**) Schematics demonstrating BCL-2 family member interactions controlling the intrinsic pathway of apoptosis. (**A**) Apoptosis through activator binding of pore formers and (**B**) primed phenotype (**C**) binding of sensitizers or BH3 mimetics displacing activators to drive apoptosis. (**D**) Schematic demonstrating the steps of BH3 profiling. (**E**) Representative flow cytometry plots of BH3 profiling and cytochrome *c* signal loss. BH3 profiling with (**F**) BIM (100 μM) and (**G**) BMF (100 μM). *n* = 5–8 mice per group. Graphed as scatterplot with bar, mean ± SEM. *P < 0.05, ***P < 0.001, 2-tailed *t* test with Welch’s correction.

Increased expression of antiapoptotic BCL-2 family members may contribute to a cell’s ability to evade apoptosis induction, but its proximity to the apoptotic threshold and relative priming state depend on the summation of interactions between antiapoptotic and proapoptotic proteins (activators, pore formers, and sensitizers) (Figure 4, A–C). We demonstrated the complexity of these changes in fibrotic fibroblasts ex vivo, showing that some antiapoptotic BCL-2 family members increase (*Bcl-2*, *Bcl-xl*) while others decrease (*Bcl-w*, *Mcl-1*), and some proapoptotic members decrease (*Bid*, *Bax*, *Bak*) (Figure 2I). To determine how these changes influenced the proximity of these fibroblasts to an apoptotic threshold, we performed BH3 profiling, a technique that determines the dynamic apoptotic potential of cells, reflecting the summative interactions of both pro- and antiapoptotic proteins, on ex vivo naive and fibrotic primary lung fibroblasts (Figure 4D). Fibroblasts were exposed to the peptides BIM (activator) and BMF (sensitizer), both of which have a strong binding affinity for BCL-2, BCL-XL, BCL-W, and MCL-1 (24). Loss of cytochrome *c* from the mitochondria was used as a surrogate for mitochondrial outer membrane permeabilization (MOMP) (Figure 4E). PDGFRα^+^ fibroblasts from fibrotic mice (10-week repetitive bleomycin and 4-week silica) were more primed than naive fibroblasts, as evidenced by a significantly greater MOMP response when exposed to BIM or BMF (Figure 4, F and G). This demonstrates that fibrotic fibroblasts are reliant on antiapoptotic BCL-2 family members to stay alive, allowing them to persist closer to the apoptotic threshold than those isolated from naive lungs persist.

### ABT-263 treatment induces fibrotic fibroblast apoptosis and reverses fibrosis in the repetitive bleomycin model of PF-ILD.

**Figure 5:**
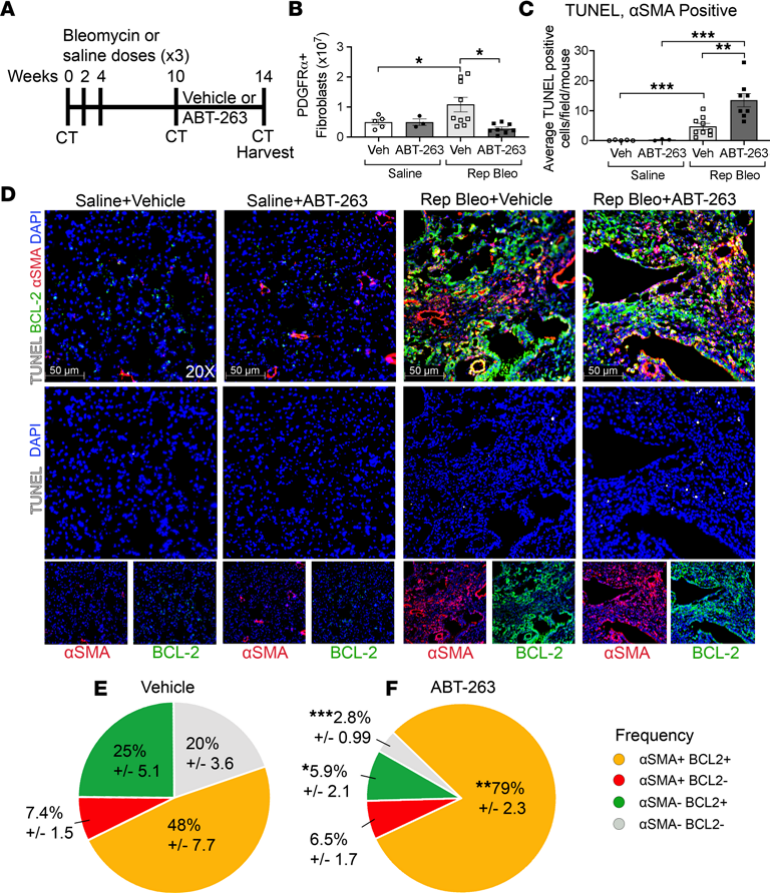
ABT-263 induces BCL-2^+^α-SMA^+^ fibrotic fibroblast apoptosis in progressive pulmonary fibrosis after repetitive bleomycin. (**A**) Schematic of repetitive dosing, ABT-263 treatment, CT, and harvesting schedule. (**B**) Quantification of Lin^–^PDGFRα^+^ fibroblast population. (**C**) Quantification of TUNEL^+^α-SMA^+^ cells per HPF and (**D**) representative images after immunofluorescence staining of lungs for BCL-2 (green), α-SMA (red), DAPI (blue), and TUNEL (white) in repetitive saline and repetitive bleomycin mice treated with vehicle or ABT-263. Pie charts demonstrating frequency of TUNEL^+^: α-SMA^+^BCL-2^+^, α-SMA^+^BCL-2^–^, α-SMA^–^BCL-2^+^, and α-SMA^–^BCL-2^–^ cells in repetitive bleomycin mice treated with (**E**) vehicle or (**F**) ABT-263. Asterisks represent significant differences in frequency between vehicle and ABT-263–treated fibrotic mice. Graphed as scatterplot with bar, mean ± SEM, or time-course line graph with mean ± SEM. *P < 0.05, **P < 0.01, ***P < 0.001, 2-tailed *t* test with Welch’s correction. Total magnification with objective 200×.

**Figure 6:**
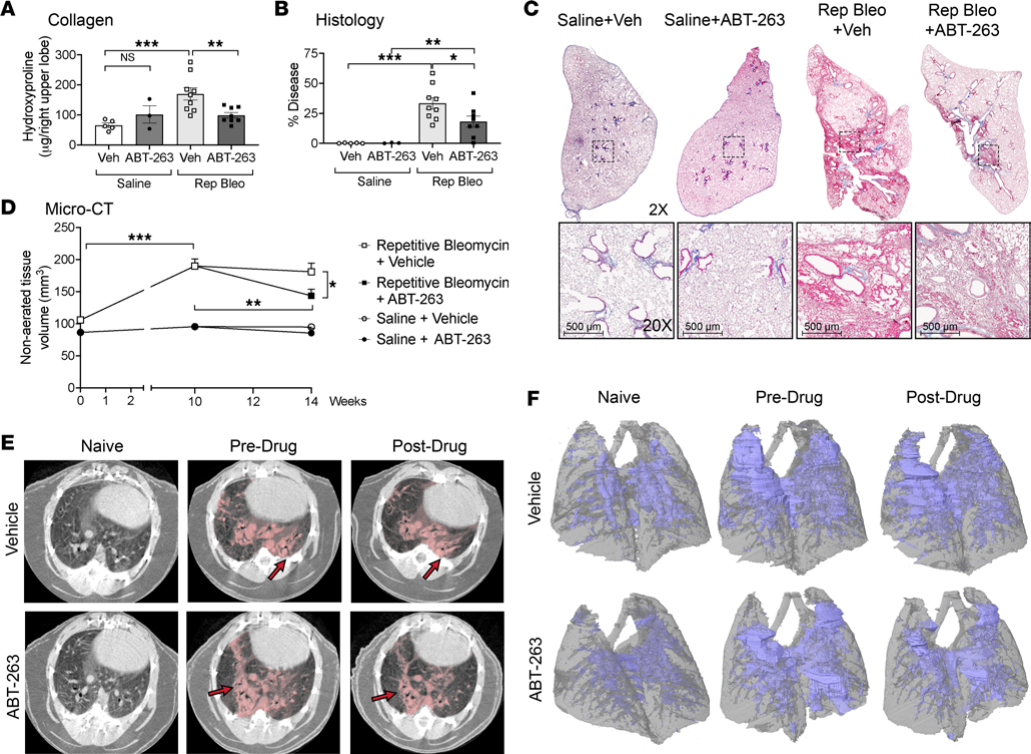
ABT-263 reverses fibrosis during therapeutic intervention in persistent, progressive fibrosis. (**A**) Hydroxyproline content of lungs, (**B**) semiquantitative histology scoring, and (**C**) representative trichrome images after vehicle or ABT-263 treatment. (**D**) Nonaerated lung volume as measured by micro-CT, with representative (**E**) axial images (red arrows indicate disease) and (**F**) 3D reconstructions (nonaerated lung in blue, aerated lung in gray). *n* = 3–9 mice per group. Graphed as scatterplot with bar, mean ± SEM, or time-course line graph with mean ± SEM. *P < 0.05, **P < 0.01, ***P < 0.001, 2-tailed *t* test with Welch’s correction. Total original magnification 2× (upper panels) and 20× (lower panels).

We have shown that in both the repetitive bleomycin and silica models of PF-ILD, fibrotic fibroblasts express antiapoptotic BCL-2 family members and are highly primed. We have also demonstrated that treatment with ABT-263 induces apoptosis in fibrotic fibroblasts to a greater extent than healthy fibroblasts in in vitro and in ex vivo PCLS. Therefore, we sought to determine if ABT-263 treatment in vivo would induce fibrotic fibroblast apoptosis and reduce fibrosis. Ten weeks after the initial instillation of bleomycin and the development of persistent fibrosis, we therapeutically treated mice with ABT-263 for 28 days (Figure 5A). ABT-263 significantly reduced PDGFRα^+^ fibroblast numbers, as measured by flow cytometry, compared with fibrotic mice receiving vehicle (Figure 5B), and did not reduce fibroblast numbers in saline-instilled controls. Additionally, within saline or repetitive bleomycin groups, treatment with ABT-263 did not alter epithelial cell (EPCAM^+^), endothelial cell (CD31^+^), or leukocyte (CD45^+^) numbers (Supplemental Figure 7). To verify that the reduction in fibroblast number was due to fibroblast apoptosis, we performed TUNEL staining on lung sections co-immunostained for α-SMA and BCL-2. We found that in fibrotic mice, ABT-263 significantly increased the number of TUNEL^+^ cells compared with vehicle controls (15.6 vs. 7.91 cells/high-powered field [HPF], respectively, *P* = 0.015), which was driven by a significant increase in α-SMA^+^TUNEL^+^ cells (Figure 5, C and D). ABT-263–treated fibrotic mice had higher proportions of TUNEL^+^ cells that were α-SMA^+^ (85.4% vs. 55.1%, *P* = 0.0035) and α-SMA^+^BCL-2^+^ (79.0% vs. 47.7%, *P* = 0.0035) compared with vehicle (Figure 5, E and F). Notably, saline-treated mice had very few TUNEL^+^ cells, which did not increase after ABT-263 treatment (0.68 vs. 0.60, *P* = 0.65, Figure 5D). We further demonstrated that treatment with ABT-263 resulted in a significant reduction in total lung collagen (Figure 6A), COL1 staining (Supplemental Figure 8, A–D), histologic appearance of fibrosis (Figure 6, B and C), and nonaerated lung volume by micro-CT (Figure 6, D–F).

### ABT-263 targets profibrotic fibroblast populations and reduces fibrosis in the murine silicosis model of PF-ILD.

**Figure 7:**
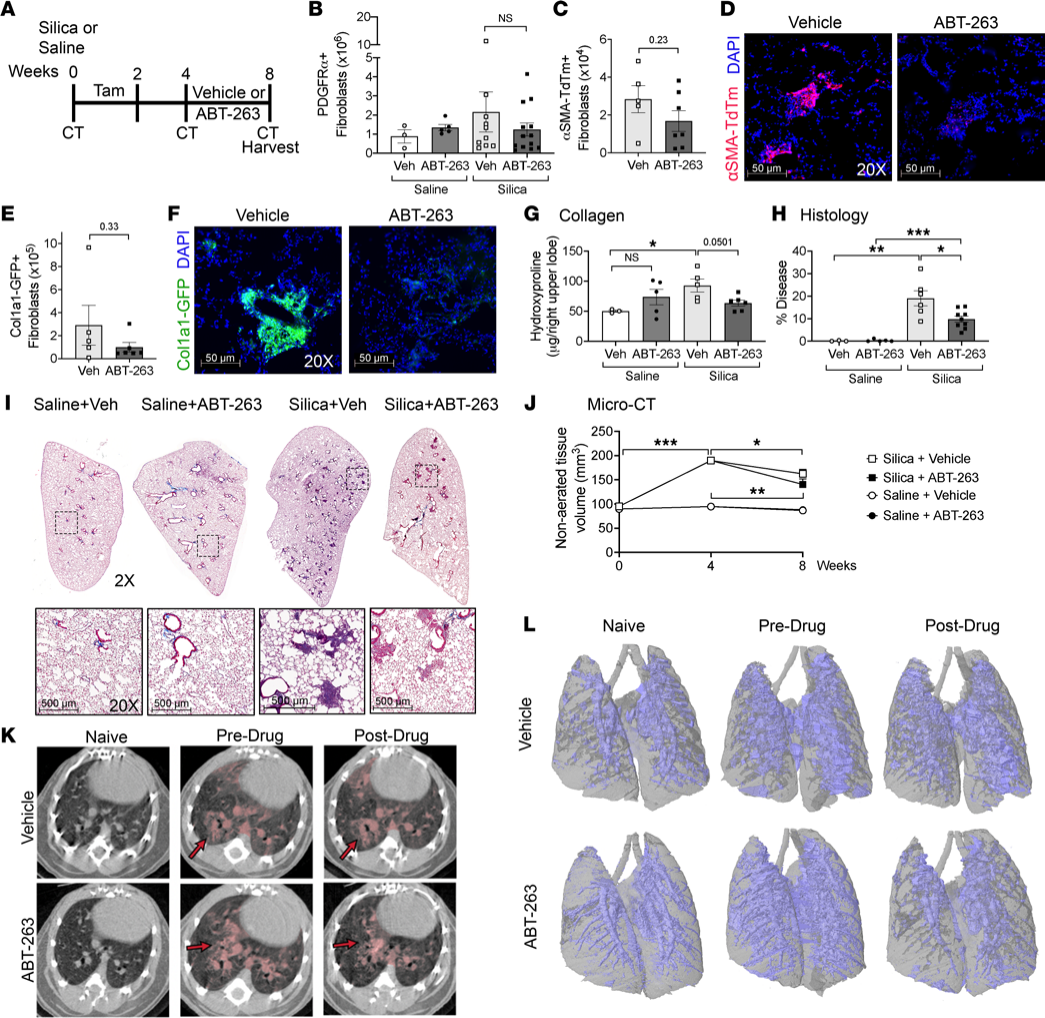
ABT-263 reduces fibrotic fibroblasts in silicotic nodules and reverses fibrosis in silicosis. (**A**) Schematic of silica treatment, tamoxifen dosing, ABT-263 treatment, and harvesting schedule. Quantitation of (**B**) PDGFRα^+^ (**C**) α-SMA TdTm^+^, and (**E**) Col1a1-GFP^+^ fibroblasts. (**D** and **F**) Representative immunofluorescence images of frozen sections (*n* = 2 per group): α-SMA TdTm^+^ (red), Col1a1-GFP^+^ (green), and DAPI (blue). (**G**) Hydroxyproline content of lungs, (**H**) semiquantitative histology scoring, and (**I**) representative trichrome images after vehicle or ABT-263 treatment. (**J**) Nonaerated lung volume as measured by micro-CT, with representative (**K**) axial images (red arrows indicate disease) and (**L**) 3D reconstructions (nonaerated lung in blue, aerated lung in gray). *n* = 3–13 mice per group. Graphed as scatterplot with bar, mean ± SEM, or time-course line graph with mean ± SEM. *P < 0.05, **P < 0.01, ***P < 0.001, 2-tailed *t* test with Welch’s correction. Total magnification with objective 200×, 2× (upper panels), 20× (lower panels).

We next validated our findings in murine silicosis as a second model of PF-ILD. Four weeks after intratracheal instillation of silica and the establishment of persistent fibrosis, we began therapeutic treatment with ABT-263 for 28 days (Figure 7A). We utilized Acta2-Cre-ERT2 TdTomato (α-SMA TdTm^+^) mice to lineage trace α-SMA^+^ fibroblasts and Col1a1-GFP mice to track collagen-producing fibroblasts. Quantitation of total lung PDGFRα^+^ fibroblasts by flow cytometry resulted in a 1.74-fold decrease after treatment with ABT-263 compared with mice with silicosis treated with vehicle (*P* = 0.43) (Figure 7B). There was a trend toward an overall decrease in the α-SMA TdTm^+^ and Col1a1-GFP^+^ subpopulations, 1.68-fold and 2.90-fold, respectively (Figure 7, C and E). However, examination of immunofluorescence imaging of frozen sections showed a striking absence of α-SMA TdTm^+^ and Col1a1-GFP^+^ populations within the silicotic nodules (Figure 7, D and F). At the time points examined, silicosis-associated fibrosis was patchy, with an overall disease burden of the lung of only approximately 10%–20%, with 80%–90% of the lung remaining nonfibrotic. Eight weeks after silica exposure, PDGFRα^+^ fibroblasts numbers increased 1.99-fold over naive lungs (Supplemental Figure 9A). The 1.74-fold reduction observed after ABT-263 treatment and loss of fibroblasts within the silicotic nodules indicate the effectiveness of ABT-263 in reducing fibrosis-associated fibroblasts from the fibrotic areas of the lung. Further examination using the 2 reporter lines demonstrated the same affect. α-SMA TdTm^+^ fibroblasts in the lungs increased 1.99-fold, and Col1a1-GFP^+^ cells increased 3.25-fold 8 weeks after silica exposure (Supplemental Figure 9, B and C). Treatment with ABT-263 resulted in a 1.68-fold and 2.90-fold reduction, respectively, supporting a targeted treatment effect on the expanding fibrotic fibroblast population. Similar to treatment in the repetitive bleomycin model, we did not detect any difference in the epithelial, endothelial, and leukocyte populations after ABT-263 treatment (Supplemental Figure 10). We found a decrease in fibrosis after treatment with ABT-263 compared with fibrotic mice treated with vehicle, with a reduction in lung collagen levels (*P* = 0.0501, Figure 7G), decreased COL1 staining (Supplemental Figure 8, E–H), a significant improvement in lung disease burden by modified stereology scoring (*P* = 0.039, Figure 7, H and I), and a significant decrease in nonaerated lung tissue volume compared with the predrug time point (*P* = 0.005) (Figure 7, J–L). We measured a reduction in nonaerated lung volume in the vehicle-treated fibrotic mice compared with the pretreatment time point, which we have previously observed between 4 and 8 weeks in prior silica studies (data not shown). We speculate that this may result from an acute alveolitis occurring early in disease, which resolves by week 8, a result confirmed by Dekoster et al., who also showed that nonaerated lung volume peaked 1 week after silica instillation, with a very slight but not statistically significant decrease between weeks 4 and 7, followed thereafter by a steady increase (32).

### Serum proteome analysis identifies biomarkers of antifibrotic effects of ABT-263.

**Figure 8:**
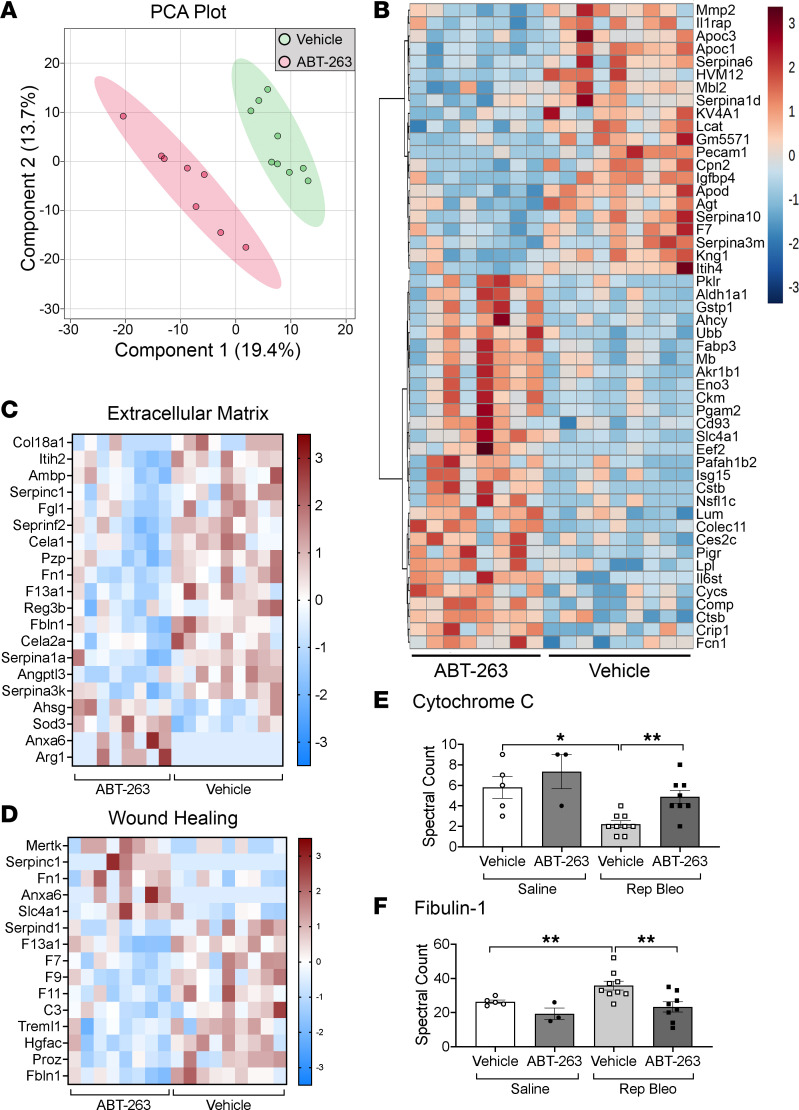
Serum proteomic analysis reveals potential biomarkers of ABT-263 antifibrotic activity. (**A**) PCA of global proteomic patterns between vehicle- and ABT-263–treated fibrotic mice. (**B**) Heatmap of normalized signal shows translational differences between vehicle and ABT-263 treatment. Heatmap of differentially abundant proteins in the enrichment pathways and associated proteins for (**C**) ECM and (**D**) wound healing. Spectral counts of (**E**) cytochrome *c* and (**F**) fibulin-1 by mass spectrometry analysis. *n* = 8–9/group. Graphed as scatterplot with bar, mean ± SEM. *P < 0.05, **P < 0.01, 2-tailed *t* test with Welch’s correction.

To determine if serum biomarkers could be identified that reflect fibroblast death and the antifibrotic activity observed after ABT-263 treatment, we performed mass spectrometry analysis on serum from repetitive bleomycin-induced fibrotic mice treated with ABT-263 and vehicle controls (Figure 5A). Principal component analysis (PCA) of highly variable proteins partitioned samples by the presence of fibrosis along the PC1 axis and treatment with ABT-263 along the PC2 axis (Figure 8A and Supplemental Figure 11). Pathway analysis of 151 differentially expressed proteins between repetitive bleomycin mice treated with vehicle or ABT-263 (Figure 8B) revealed enrichment for pathways associated with ECM reorganization (*P* = 3.66 × 10^–6^, 5.28-fold) and wound healing (*P* = 7.45 × 10^–9^, 16.24-fold) (Figure 8, C and D, and Supplemental Table 3). Cytochrome *c*, a key mediator of apoptosis whose level in the serum reflects cell death (33) that is released from cells after ABT-263 binding of antiapoptotic proteins (34), was elevated in the serum of ABT-263–treated fibrotic mice compared with fibrotic vehicle controls (Figure 8E). We also examined biomarkers that have been shown to reflect aberrant fibrogenesis in patients with PF-ILD (MMP-1, MMP-7, TIMP, CCL18, OPN, periostin, YKL40, fibulin-1, sLOXL2, ADAM12) (35) and found that fibulin-1 was significantly increased in mice with persistent and progressive fibrosis and decreased back to levels of saline-instilled controls after treatment with ABT-263 (Figure 8F).

## Discussion

PF-ILDs are a heterogenous group of diseases that can arise after varying pathogenic insults. However, they all share a central and common feature of progression with accumulation and persistence of profibrotic fibroblasts within the injured and architecturally distorted alveolar walls and airspaces (36). Unlike normal, homeostatic wound repair, where fibroblasts undergo apoptosis and are cleared at the completion of the repair process (5, 12, 37), fibrotic lung fibroblasts develop resistance to apoptosis, a phenomenon that is thought to result in their persistence and ability to further synthesize fibrotic scar tissue (5, 38). Here, we demonstrate that increased expression of antiapoptotic BCL-2 family members occurs in 2 human PF-ILDs and in 2 preclinical models of persistent fibrosis. We also show that despite heterogeneity in the mechanisms of fibrosis initiation between silicosis and repetitive bleomycin (29, 39, 40), the fibrotic fibroblasts are targeted therapeutically to undergo apoptosis with the BH3 mimetic ABT-263, resulting in the reversal of established fibrotic disease.

As fibrotic fibroblasts employ antiapoptotic BCL-2 family proteins to stay alive through the sequestration of proapoptotic activators, their priming status shifts, allowing them to become exquisitely sensitive to the effects of BH3 mimetics (24, 27, 41, 42). BCL-2 inhibition with the BH3 mimetics have demonstrated efficacy in the single-dose bleomycin model to inhibit the development of fibrosis (if given during the inflammatory phase) and hasten spontaneous resolution of fibrosis (if given during the fibrotic phase) (26, 43, 44). However, because single-dose bleomycin is a model of homeostatic fibrosis resolution (6, 8, 12), it is a suboptimal model for studying fibroblast accumulation and resistance to apoptosis in the context of nonresolving lung disease, thus prompting our use of repetitive bleomycin and silicosis as models of persistent, progressive pulmonary fibrosis. Similar to the effect seen in dermal fibroblasts in a persistent model of bleomycin-induced scleroderma (27), we found ABT-263 induced apoptosis of α-SMA^+^BCL-2^+^ fibrotic fibroblasts in the lungs of mice after repetitive bleomycin. Additionally, ABT-263 reduced the presence α-SMA TdTm^+^ and Col1a1-GFP^+^ fibrotic fibroblasts within silicotic nodules. Examination of α-SMA^+^ fibroblasts after ABT-263 treatment of human healthy and IPF PCLS supported enhanced sensitivity and apoptosis that we predicted would occur with the coexpression of antiapoptotic BCL-2 family members in IPF lung tissue.

We sought to determine a mechanism for the preferential targeting of fibrotic fibroblasts by ABT-263. By examining gene and protein expression of antiapoptotic BCL-2 family members and integrating BH3 profiling using ex vivo–isolated primary fibroblasts from naive and fibrotic lungs, we found that fibrotic fibroblasts had increased expression of *Bcl-xl*, and reduced expression of *Bax*, *Bak*, and *Bid*, with a trend toward increased *Bcl-2* expression, along with increased sensitivity to BIM and BMF. The decreased expression of the proapoptotic genes *Bax*, *Bak*, and *Bid* did not result in a reduced priming state because changes in the relative proportions of pro- and antiapoptotic members does not alter the sensitivity of a cell to the apoptotic threshold in a 1:1 relationship. For example, a single BCL-XL molecule has been shown to inhibit up to 4 BAX molecules (45). This highlights the importance of BH3 profiling, which reflects the summative interactions of all antiapoptotic and proapoptotic members. Therefore, even though some proapoptotic members were downregulated in fibrotic lungs, our data suggest that the antiapoptotic members are sequestering sufficient proapoptotic members as suggested by both the BH3 profiling and their enhanced response to ABT-263.

Taken together, the combined phenotype indicates that fibrotic fibroblasts have both increased resistance to apoptosis and a more primed phenotype compared with fibroblasts from a naive lung, thus predicting their increased sensitivity to BH3 mimetics that target the antiapoptotic proteins they employ. This was supported in vivo, as saline control mice receiving ABT-263 or vehicle had similar numbers of fibroblasts and TUNEL^+^ cells. Regulation of BCL-2 family member expression and sensitivity to ABT-263 in fibroblasts of fibrotic lungs may be due to mechanotransduction signaling and senescence. Lagares et al. demonstrated that dermal fibroblasts cultured on stiff substrates have increased expression of *Bcl-2* and *Bcl-xl*, increased fibroblast priming, and increased susceptibility to ABT-263 compared with fibroblasts cultured on soft substrates (27). We hypothesize that the increased stiffness within fibrotic lungs (46) may contribute to the primed phenotype we have described in the fibrotic fibroblasts, thus rendering them more susceptible to apoptosis after treatment with ABT-263. Our ability to profile the apoptotic potential of ex vivo–isolated cells, never exposed to the stiffness of tissue culture plastic, provide a potentially novel insight into the accurate in vivo apoptotic potential of these cells. An additional mechanism of action to consider is senescence and its associated mitochondrial dysfunction (47). ABT-263 is also known to function as a senolytic (48) and has been shown to be successful in eliminating senescent cell populations in several preclinical models, including radiation-induced lung fibrosis (49). This may contribute to its specificity of action against senescent fibrotic fibroblasts in PF-ILD lungs (50, 51). We were unable to passage senescence-associated β-galactosidase^+^ primary lung fibroblasts from 28-week repetitive bleomycin mice as they had slow doubling times that eventually halted, suggesting a senescent phenotype at this time point.

It is worth exploring the idea that the priming status of fibroblasts may change over time as fibrosis progresses. While we observed similar patterns of expression of antiapoptotic members (BCL-2, BCL-XL, BCL-W, MCL-1) at 10 and 28 weeks after initiating bleomycin-induced injury and at 4 and 24 weeks after silica, this does not fully account for the complex interactions between the members of the Bcl-2 family, including the availability of activators, sensitizers, and pore formers, which may vary over time. A further comprehensive study in the mouse models of persistent fibrosis and availability of human tissue with interstitial lung abnormalities would be needed. Additionally, variability in the ratio between BCL-2 family members could be influenced by the broad spectrum of injurious stimuli that are associated with PF-ILDs. We observed a lower proportion of α-SMA^+^ myofibroblasts with coexpression of BCL-2 in patients with silicosis compared with those with IPF. We hypothesize that because silicosis is associated with persistent inflammation and injury to the lungs (52), the ratios of antiapoptotic BCL-2 family members may be different than for α-SMA^+^ fibroblasts associated with IPF.

BH3 mimetics, including ABT-263, were originally developed as anticancer agents in response to the observation that some cancer cells overexpress BCL-2 and BCL-XL (25). ABT-263 is orally bioavailable and has been studied in multiple clinical trials for chronic lymphocytic leukemia, lymphoma, and solid tumors (25), and its primary limiting toxicity has been thrombocytopenia because platelets rely on BCL-XL to survive (34). As such, the potential effect on other cell populations beyond fibrotic fibroblasts, particularly given the predominant senescent phenotype of epithelial cells in PF-ILDs (53), must also be considered. Immunostaining for BCL-2 family members was apparent in α-SMA^–^ cells in the lung (Figures 1 and 2 and Supplemental Figures 1 and 4), which resulted in apoptosis of some α-SMA^–^ cells after treatment with ABT-263 (Figures 3 and 5). However, taken together, we found no significant loss in individual lineage-positive epithelial, endothelial, or leukocyte numbers between vehicle and ABT-263 treatment, in naive or fibrotic lungs. Multiple cell types are involved in the pathogenesis of fibrotic lung disease, including epithelial, endothelial, and leukocyte populations (53–55). Early apoptosis of the alveolar type 2 epithelial cells (AEC2s) is an essential part of pulmonary fibrosis development (56). The AEC2s in IPF have been shown to have increased apoptosis compared with healthy controls as part of fibrosis initiation. During established fibrosis, alveolar epithelial cells from IPF lungs are characterized as having less BCL-2 and more BAX compared with those in a healthy lung (57), and BCL-XL overexpression has been shown to protect alveolar epithelial cells from cell death after bleomycin (58). This suggests a different priming state when compared with fibrotic fibroblasts. Gu et al. found that ABT-199 (a BH3 mimetic that specifically inhibits BCL-2) only slightly enhanced AEC2 apoptosis compared with vehicle controls when given during bleomycin-induced fibrosis development (days 12–21) (43). Profibrotic macrophages have also been implicated in the pathogenesis of fibrotic lung disease (28, 59). The mitochondria from airspace macrophages isolated from patients with IPF have 4-fold higher expression of BCL-2 compared with controls, while *Bcl-2* deletion in macrophages or inhibition with ABT-199 has been shown to prevent fibrosis development after both bleomycin- and asbestos-induced fibrosis and accelerate spontaneous resolution in mice after single-dose bleomycin (43). Platelets highly express BCL-XL and are susceptible to death after ABT-263 treatment (34). However, platelets from patients with IPF have been shown to have increased activity compared with healthy patients (60), and their inhibition was antifibrotic in a mouse model of pulmonary fibrosis (61). Pulmonary hypertension, a common comorbidity in PF-ILD, has a prevalence of approximately 30%–50% in patients with IPF (62). In a rat model of pulmonary hypertension, ABT-263 was shown to reverse pulmonary vascular remodeling through apoptotic targeting of pulmonary artery smooth muscle cells (63). Taken together, this body of preclinical data suggest that the targeting of multiple cell populations (fibroblasts, AEC2s, macrophages, platelets, and vascular smooth muscle cells) with BH3 mimetics results in beneficial fibrosis outcomes. The specificity of the BH3 mimetics to target fibrotic cells is highly dependent upon the priming state of each cell population, which may not only be disease specific but also change temporally from disease initiation though progression.

Biomarkers provide useful information regarding diagnosis, prognosis, or treatment response for many diseases, including PF-ILDs (35, 64–66). However, the preclinical and clinical development of novel therapeutic candidates is still hindered due to a lack of a suitable biomarker that provides an early indication of drug efficacy and patient benefit (67). We performed proteomic analysis of serum to identify biomarkers that might reflect the antifibrotic treatment response of ABT-263. Recently the PROFILE study found significant differences in ECM neo-epitopes in serum from patients with IPF compared with controls that also correlated with disease progression (66). While our mass spectrometry analysis did not assess ECM-derived neo-epitopes, it revealed that biologically relevant pathways associated with ECM organization (*P* = 3.66 × 10^–6^) and wound healing (*P* = 1.56 × 10^–5^) were significantly enriched after ABT-263 treatment of fibrotic mice. We observed a significant decrease in the matrix proteins fibronectin (*P* = 0.0045), fibulin-1 (*P* = 0.0070), and fibrinogen-like protein 1 (*P* = 0.0350). Fibulin-1, a glycoprotein produced by lung fibroblasts that is increased in the serum of patients with IPF and correlates with disease severity (65, 68), was decreased in ABT-263–treated fibrotic mice. Similar to a recent publication by Mathai et al., which examined serum from healthy controls and patients with IPF (65), we observed significant differences in serum levels between naive and fibrotic vehicle-treated mice of fibulin-1 (Fbln1, *P* = 0.026), S100A9 (*P* = 0.0269), brain acid soluble protein 1 (Basp1, *P* = 4.74 × 10^–6^), tetranectin (Clec3B, *P* = 2.44 × 10^–4^), kininogen-1 (Kng1, *P* = 6.3 × 10^–4^), apolipoprotein A-IV (ApoA4, *P* = 4.91 × 10^–3^), apolipoprotein A-II (ApoA2, *P* = 0.026), haptoglobin (Hp, *P* = 0.031), CRK-like-protein (Crkl, *P* = 0.0426), and lipopolysaccharide binding protein (Lbp, *P* = 0.0279). We found that C1qb was higher in serum of our fibrotic mice compared with saline-treated mice. C1q has been previously shown to activate pulmonary fibroblasts in a model of murine silicosis (69), and increased expression in lung tissue has been associated with a poor prognosis in IPF (70). Finally, we found that cytochrome *c*, a serum marker of apoptosis (33, 34) was higher in ABT-263–treated fibrotic mice. Together, cytochrome *c* and fibulin-1 have potential as serum biomarkers reflecting both the apoptotic and antifibroblast activity of ABT-263. While no single serum biomarker has currently been shown to accurately predict the presence or absence of pulmonary fibrosis, serum from persistently fibrotic mice after repetitive bleomycin show many similar changes to those seen in human fibrotic lung disease, further supporting its strength as a model of PF-ILD.

In summary, we found that fibrotic fibroblasts exhibited a primed phenotype, making antiapoptotic BCL-2 family members relevant therapeutic targets in PF-ILDs. We successfully reduced fibrotic fibroblast numbers and reversed pulmonary fibrosis in mice with persistent, progressive disease after therapeutic intervention with ABT-263. We propose that cytochrome *c* and fibulin-1 serve as preclinical biomarkers that can be followed in future studies as surrogates for drug efficacy. While further studies are required to dissect the cellular specificity of ABT-263 and its effects on senescent cells in persistent, progressive preclinical models, ABT-263 has now been shown to be effective in reducing fibrosis development in multiple models of fibrosis, including the skin (27). Additionally, because it has already undergone phase II clinical trials, ABT-263 is a safe and relevant potential therapeutic to be considered as a treatment for patients with PF-ILD.

## Methods

### Animals and models of persistent fibrosis.

Male C57BL/6N mice (Envigo), Acta2-Cre-ERT2 TdTomato (α-SMA TdTm^+^) mice (Stijn De Langhe, Mayo Clinic; ref. 71), and Col1a1-GFP^+^ mice (David Brenner, University of California, San Diego, La Jolla, California, USA; ref. 72), aged 8–16 weeks, were maintained in a pathogen-free environment with 12-hour light/12-hour dark cycles and full access to food and water. To express the TdTomato reporter, α-SMA TdTm^+^ mice received 4 intraperitoneal injections of tamoxifen (0.25 mg/g body weight in corn oil, MilliporeSigma) days 4–21 after silica. Persistent, progressive pulmonary fibrosis was initiated with 3 biweekly intratracheal instillations of bleomycin (1 U/kg body weight) (Fresenius Kabi) or after a single intratracheal instillation of crystalline silica (0.2 mg/g body weight, particle size 1–3.5 μm, Nanostructured & Amorphous Materials Inc) as previously described (29, 73). Control animals received intratracheal instillation of saline. We will refer to mice based on their fibrotic exposure (repetitive bleomycin or silica) and time from the initial instillation. Five to 10 mice were instilled for each experimental treatment condition based on a power analysis to detect significant differences in fibrosis.

### Immunofluorescence staining and protein analysis.

Immunofluorescence staining was performed on 10% formalin-fixed, paraffin-embedded or frozen OCT-embedded (Thermo Fisher Scientific) sections (12, 29, 74). Briefly, sections were immunostained for anti–α-SMA (1:500, MilliporeSigma, clone 1A4), COL1 (1:250, MilliporeSigma, 765P), and anti–BCL-2 family member proteins — BCL-2 (1:500, Cell Signaling Technology, 3498), BCL-XL (1:500, Cell Signaling Technology, 2764), BCL-W (1:500, MilliporeSigma, 4502627), MCL-1 (1:500, Cell Signaling Technology, 94296), BAX (1:500, Cell Signaling Technology, 2772), or BAK (1:500, Cell Signaling Technology, 12105) — overnight at 4°C followed by incubation with fluorescently tagged goat anti-mouse–A647 (A-21236), donkey anti-rabbit–A555 (A-31572), or goat anti–chicken–A647 (A-21449) secondary antibodies at 1:100 dilution (Invitrogen). Negative controls were nonimmune IgG (2729 and 5415, 1:500, Cell Signaling Technology) with secondary or secondary alone (Supplemental Figure 12). To validate BCL-2 antibody specificity for the BCL-2 family of proteins, we performed Western blot analysis of healthy human fibroblasts (Supplemental Figure 13). To detect apoptotic cells, TUNEL staining was performed prior to antibody staining per manufacturer instructions (Promega). Images were acquired on a Zeiss Axioplan 2 epi-fluorescence microscope and analyzed with Axiovision software (Zeiss). Semiquantitative assessment of BCL-2 family member and α-SMA^+^ cell costaining was determined as 0 (0%), 1 (1%–33%), 2 (34%–66%), or 3 (67%–100%) in a blinded manner on 10 images/sample. α-SMA^+^ cells not surrounding vessels or airways were considered fibrotic fibroblasts, and α-SMA^+^ cells surrounding airway walls or blood vessels were excluded. Prior to immunostaining, PCLS were washed twice in PBS, fixed in 4% paraformaldehyde for 20 minutes, permeabilized in PBS with 1% Triton X-100 (PBS-Triton) for 10 minutes, and blocked with 10% normal horse serum in PBS-Triton for 2 hours. Sections were immunostained for cleaved caspase-3 (9661, 1:500, Cell Signaling Technology) and α-SMA in 10% normal horse serum in PBS-Triton overnight at 4°C with gentle shaking. Sections were washed 3 times for 45 minutes in PBS-Triton, prior to incubation with fluorescently tagged goat anti-mouse–A647 (A-21236) and donkey anti-rabbit–A555 (A-31572) at a 1:200 dilution (Invitrogen) in 10% normal horse serum in PBS-Triton overnight at 4°C with gentle shaking. Samples were placed in 60% glycerol with 2.5 M fructose in distilled H_2_O for 10 minutes, then coverslipped using two 0.12 mm separators (Grace Bio-Labs) and imaged on a Zeiss confocal microscope. Five images from each of the PCLS were counted for colocalized cleaved caspase-3 and α-SMA^+^ fibroblasts. Negative controls were stained with nonimmune IgG (1:500, Cell Signaling Technology) with secondary or secondary alone (Supplemental Figure 14).

### Human lung tissue and PCLS.

Healthy human lung tissue was obtained from Donor Alliance, and IPF tissue was obtained from lung biopsies or explanted lungs from confirmed cases of IPF (IRB 11-1664) as described previously (74). Tissue from patients with silicosis was obtained after lung transplant as described previously (75). For the generation of PCLS, freshly explanted healthy or IPF lungs were kept on ice and processed within 24 hours. Peripheral sections of lungs were inflated with low–melting point agarose (MilliporeSigma) by cannulation of a visible bronchus; then 6 × 6 × 6 mm cubes were cut from the lung, embedded in low–melting point agarose, and sectioned using a vibratome (Leica). We cultured 300 μm slices in Eagle minimal essential medium (EMEM, Lonza) with 10% heat-inactivated fetal bovine serum (FBS, Atlanta Biologicals) and 1% penicillin/streptomycin/l-glutamine with or without ABT-263 (5 μM, Medchemexpress) for 24 hours.

### Primary lung fibroblast culture.

Primary lung fibroblasts were isolated from naive and silica-exposed (8 weeks after instillation) mice and healthy and IPF human lungs as described (13, 74, 76). Briefly, freshly explanted lung tissue was minced into 1–2 mm^3^ sections, mouse samples were cultured in Dulbecco’s modified Eagle medium (DMEM, Lonza) with 10% FBS and 1% penicillin/streptomycin, and human fibroblasts were cultured in EMEM with 10% FBS and 1% penicillin/streptomycin/l-glutamine. Fibroblasts were used between passages 3 and 8.

### Flow cytometry.

Single-cell suspensions were obtained from perfused, enzymatically dispersed lungs using a digestion mixture of collagenase (450 U/mL), dispase (5 U/mL), and elastase (4 U/mL) and incubated at 37°C for 25 minutes, followed by a secondary digestion in 0.1% trypsin-EDTA with 0.33 U/mL DNase I for 20 minutes at 37°C (12). Cells were stained with fluorescently tagged monoclonal antibodies against CD45 (17-0451-82, 56-0451-82), CD31 (17-0311-82), CD326/EPCAM (17-5791-82, 25-5791-80), and PDGFRα/CD140a (25-1401-82) (1:100, Thermo Fisher Scientific). Cell analysis data were acquired with the LSRFortessa (BD Biosciences) and analyzed with FlowJo 2 software (Tree Star).

### BH3 profiling.

Intracellular BH3 profiling was performed as described (77) on primary lung fibroblasts. Single-cell suspensions were obtained from enzymatically dispersed naive and fibrotic lungs. Lin^–^ cells were column enriched by incubating with CD45, CD31, and CD326 MicroBeads and purified off LS columns per manufacturer’s instructions (Miltenyi Biotec). Lin^–^ cells were stained with Live/Dead (1:100, L34965, Invitrogen) and anti-PDGFRα, -CD31, -CD45, and -EPCAM (1:100, Invitrogen, Thermo Fisher Scientific) for 30 minutes. Cells were permeabilized with 0.002% digitonin in MEB2 buffer (150 mM mannitol, 10 mM HEPES-KOH, 150 mM KCl, 1 mM EGTA, 1 mM EDTA, 0.1% BSA, 5 mM succinate in sterile distilled H_2_O at a pH of 7.5) and exposed to BIM or BMF (100 μM, New England Peptides), DMSO, or 25 μM alamethicin (Enzo) for 60 minutes. Cells were fixed with 4% paraformaldehyde for 10 minutes, then solution neutralized (1.7 M Tris base, 1.25 M glycine, pH 9.1), stained with anti–cytochrome *c* (1:40, 11-6601-82, Invitrogen) in intracellular staining buffer (2% Tween 20 and 0.1 g/mL BSA in PBS) overnight at 4°C followed by FACS analysis on the LSRFortessa, and analyzed with FlowJo 2 software. Depolarization (% cytochrome *c* loss) was determined by measuring median fluorescence intensity (MFI) of the FITC (cytochrome *c*) gate and adjusting for positive and negative controls: depolarization = 1 – (MFI_sample_ – MFI_alamethicin_)/(MFI_DMSO_ – MFI_alamethicin_) (77).

### qPCR, drug treatment, and measurement of apoptosis.

RNA was isolated from enriched Lin^–^ cells or in vitro fibroblasts using the RNeasy kit per manufacturer guidelines (QIAGEN). qPCR was performed using Quantitect reverse transcription kit (QIAGEN). Primers for the following were used (Supplemental Table 4): *Bcl-2*, *Bcl2l1* (*Bcl-xl*), *Bcl2l2* (*Bcl-w*), *Mcl-1*, *Bax*, *Bak1*, *Bid*, *Bcl2l11* (*Bim*), and Acta2 (α-SMA) (Integrated DNA Technologies). Data were analyzed using the ^ΔΔ^Ct method and normalized to *Gusb* (Integrated DNA Technologies).

Cells were treated with ABT-263 (Medchemexpress) in DMEM (5 μM, mouse) or in EMEM (1 μM, human) with 0.25% FBS for the final 24 hours or 1 μM staurosporine (MilliporeSigma) for the final 5 hours. After a total of 72 hours, caspase-3 and -7 activity was measured by luminescence assay (Caspase-Glo 3/7, Promega) and read on a Biotek FLX800 microplate fluorescence reader. A concentration of 5 μM ABT-263 was used for mouse samples as 1 μM did not demonstrate increased apoptosis (data not shown).

### In vivo drug treatment and assessment of disease.

Beginning after fibrosis was established (4 weeks after silica or 10 weeks after the initial bleomycin dose), mice were administered 100 mg/kg ABT-263 in DMSO and sterile corn oil or vehicle (sterile corn oil with DMSO) by daily oral gavage for 28 days. Mice underwent micro-CT scanning prior to the induction of fibrosis, prior to treatment with ABT-263, and at study termination (Skyscan 1276, Bruker MicroCT). Images were acquired at 35 μm resolution, with x-ray tube voltage 50 kV, current 500 A, exposure time 900 ms, with 0.5 mm aluminum filter and a 0.7° rotation step. Scans were reconstructed as described (29). Fibrosis was assessed by measuring nonaerated lung tissue volume by micro-CT, lung collagen quantified by hydroxyproline in the upper right lobe (12, 29), and semiquantitative stereology-based point-counting assessment of fibrosis in the left lung of formalin-fixed, paraffin-embedded, 5 μm sections stained with Masson’s trichrome and imaged by Aperio Scanning (Leica Biosystems) (74). PDGFRα^+^ fibroblast numbers were measured by flow cytometry (12).

### Liquid chromatography mass spectrometry analysis of serum.

Whole blood was obtained from the heart and allowed to clot followed by a serum separation spin (centrifuge 6,000*g* for 20 minutes at 4°C). The protein concentration for each serum sample was determined (Pierce A660 Protein Assay, Thermo Fisher Scientific), and proteolytic digestion, sample preparation, and peptide recovery were carried out according to the FASP protocol (78). Global proteomics was performed on an LC-MS Fusion Lumos Tribrid mass spectrometer (Thermo Fisher Scientific) operating in the positive ion mode (78). Raw data were searched using an in-house Mascot server (Version 2.5, Matrix Science) through Proteome Discoverer (Thermo Fisher Scientific, version 2.5). The generated data were searched against SwissProt (17,029 sequences) restricted to *Mus musculus* using version 1.1 of the CRAPome for common contaminants (79). PCA was conducted using the *factoextra* R package version 1.0.7 with intensity data for all proteins, and differential protein expression was determined. Differential protein expression was calculated by the Differential Expression analysis of quantitative Mass Spectrometry data (DEqMS) statistical method in R (version 4.2.0) using the DEqMS R package (version 1.14.0) in Bioconductor. Pathway analysis was conducted with Panther GO Biological Processes and GO Cellular Compartments with a Fisher’s exact test with Bonferroni’s correction for multiple testing (80).

### Statistics.

Data are analyzed as mean ± SEM using GraphPad Prism software (Version 8), and differences between conditions at specific time points were examined using a 1-way Brown-Forsythe ANOVA with Welch’s and Dunnett’s corrections for multiple comparisons or Welch’s *t* test. *P* < 0.05 using 2-tailed tests was considered significant. Individual animal replicates are indicated in the figure legends.

### Study approval.

All human samples were obtained in accordance with approved IRB protocol IRB 11-1664 (University of Colorado). All animal studies were approved by the National Jewish Health Institutional Animal Care and Use Committee.

## Author contributions

JCC, EFR, and DWHR participated in study conception; experimental design; data acquisition, analysis, and interpretation; and manuscript preparation. DAS, LAO, NJ, JAW, DGF, and BLE participated in data acquisition and analysis and manuscript preparation.

## Supplementary Material

Supplemental data

## Version 1 02/08/2023

Electronic publication
