# Supplementary material for: Inhibition of antiapoptotic BCL-2 proteins with ABT-263 induces fibroblast apoptosis, reversing persistent pulmonary fibrosis
Source: JCI Insight. 2023 Feb 8;8(3):e163762. doi: 10.1172/jci.insight.163762 (PMC9977433; doi:10.1172/jci.insight.163762)
Supplement: Supplemental data [file jciinsight-8-163762-s095.pdf]

Characteristics	Healthy (n=5)	IPF (n=8)	Silicosis (n=8)*
Male sex, n (%)	2 (40%)	5 (62.5%)	8 (100%)
Age, yr, mean +/- SD	63+/- 10.5	59 +/- 9.8	65 +/- 6.8
Age Distribution n (%)			
40 to <50	1 (20%)	2 (25%)	
50 to <60	0 (0%)	2 (25%)	1 (12.5%)
60 to <70	3 (60%)	4 (50%)	4 (50%)
70 to <80	1 (20%)	0 (0%)	2 (25%)
N/A**	0 (0%)	0 (0%)	1 (12.5%)

Supplemental Table 1. Demographic data for immunofluorescent staining samples

*Samples from 4 patients were stained for each BCL-2 family member. **One silicosis patient's age was unidentifiable.

Characteristics	Healthy (n=3)	IPF (n=3)
Male sex, n (%)	3 (100%)	1 (33%)
Age, yr, mean +/- SD	57+/- 4.5	70 +/- 4.5
Age Distribution n (%)		
50 to <60	2 (67%)	0 (0%)
60 to <70	1 (33%)	1 (33%)
70 to <80	0 (0%)	2 (67%)

Supplemental Table 2. Precision cut lung slice donor demographic data

GO biological process	Expected Value	Fold Enrichment	+/-	P value
Methylglyoxal metabolic process	0.04	92.81	+	4.69E-03
L-phenylalanine catabolic process	0.06	69.61	+	1.09E-02
Erythrose 4-phosphate/phosphoenolpyruvate family amino acid catabolic process	0.06	69.61	+	1.09E-02
L-phenylalanine metabolic process	0.07	55.69	+	2.19E-02
Fructose metabolic process	0.09	53.55	+	1.28E-03
Aldehyde catabolic process	0.11	46.41	+	2.28E-03
Canonical glycolysis	0.11	43.51	+	2.98E-03
Glucose catabolic process to pyruvate	0.11	43.51	+	2.98E-03
NADH regeneration	0.11	43.51	+	2.98E-03
Blood coagulation, fibrin clot formation	0.14	41.77	+	2.27E-04
Glycolytic process through glucose-6-phosphate	0.14	36.64	+	6.11E-03
Alditol metabolic process	0.17	36.32	+	4.60E-04
Monosaccharide biosynthetic process	0.31	35.61	+	1.16E-09
Glucose catabolic process	0.14	34.81	+	7.60E-03
NAD metabolic process	0.2	34.81	+	4.33E-05
Hexose biosynthetic process	0.26	34.81	+	2.50E-07
Protein activation cascade	0.18	33.41	+	7.04E-04
Glycolytic process through fructose-6-phosphate	0.15	33.15	+	9.35E-03
Gluconeogenesis	0.23	30.45	+	9.67E-05
NADH metabolic process	0.25	27.84	+	1.67E-04
Cellular aldehyde metabolic process	0.4	27.35	+	1.44E-08
Purine ribonucleoside metabolic process	0.19	25.78	+	2.77E-02
Response to copper ion	0.2	24.86	+	3.24E-02
Cellular detoxification	0.27	21.98	+	6.26E-03
Cellular response to toxic substance	0.32	18.56	+	1.53E-02
Glutathione metabolic process	0.57	17.62	+	7.43E-06
Detoxification	0.4	17.4	+	3.01E-03
Blood coagulation	0.85	16.52	+	6.04E-09
Alpha-amino acid catabolic process	0.55	16.49	+	9.12E-05
Regulation of plasma lipoprotein particle levels	0.37	16.38	+	2.98E-02
Hemostasis	0.86	16.24	+	7.45E-09
Monosaccharide metabolic process	1.17	15.37	+	8.25E-12
Hexose metabolic process	1.05	15.26	+	3.93E-10
Primary alcohol metabolic process	0.56	14.28	+	1.72E-03
Pyruvate metabolic process	0.5	14.12	+	1.10E-02
Cellular amino acid catabolic process	0.67	13.47	+	4.58E-04
Negative regulation of endopeptidase activity	1.34	13.47	+	6.85E-11
Cellular modified amino acid metabolic process	1.38	13.05	+	1.14E-10
Cellular ketone metabolic process	0.62	12.95	+	3.44E-03
Negative regulation of peptidase activity	1.87	12.27	+	6.51E-14

Cholesterol homeostasis	0.69	11.6	+	7.49E-03
Sterol homeostasis	0.7	11.48	+	8.06E-03
Polyol metabolic process	0.73	11.03	+	1.07E-02
Alcohol metabolic process	2.21	10.88	+	1.52E-13
Response to toxic substance	0.87	10.36	+	3.71E-03
Negative regulation of hydrolase activity	2.62	10.3	+	4.53E-15
Secondary alcohol metabolic process	0.98	10.16	+	9.75E-04
Cholesterol metabolic process	0.89	10.1	+	4.51E-03
Carboxylic acid catabolic process	1.42	9.84	+	3.71E-06
Organic acid catabolic process	1.44	9.75	+	4.20E-06
Negative regulation of proteolysis	2.59	9.67	+	4.17E-13
Organic hydroxy compound transport	1.06	9.47	+	1.81E-03
Sterol metabolic process	0.96	9.42	+	7.83E-03
Cellular carbohydrate metabolic process	1.06	9.41	+	1.92E-03
Regulation of cellular ketone metabolic process	1.1	8.19	+	2.35E-02
Regulation of peptidase activity	3.18	8.17	+	4.28E-12
Regulation of lipid biosynthetic process	1.47	8.15	+	5.01E-04
Response to reactive oxygen species	1.13	7.98	+	2.87E-02
Wound healing	1.9	7.88	+	1.56E-05
Sulfur compound metabolic process	2.29	7.86	+	3.51E-07
Regulation of endopeptidase activity	2.57	7.78	+	3.16E-08
Regulation of lipid localization	1.34	7.49	+	1.39E-02
Positive regulation of lipid metabolic process	1.35	7.41	+	1.52E-02
Regulation of small molecule metabolic process	2.66	6.4	+	2.50E-05
Steroid metabolic process	1.94	6.19	+	8.40E-03
Response to oxidative stress	2.47	5.67	+	2.80E-03
Response to metal ion	2.13	5.64	+	2.11E-02
Small molecule metabolic process	10.9	5.59	+	1.41E-25
Response to inorganic substance	3.31	5.44	+	9.55E-05
Regulation of lipid metabolic process	2.77	5.41	+	1.83E-03
Response to hormone	4.25	4.48	+	7.20E-04
Cellular amide metabolic process	5.45	4.04	+	3.70E-04
Cellular lipid metabolic process	6.51	3.22	+	2.70E-02
Response to oxygen-containing compound	9.85	3.15	+	1.39E-04
Response to organic substance	18.75	2.13	+	2.93E-02
Organic substance metabolic process	53.01	1.96	+	2.65E-12
Cellular metabolic process	47.88	1.8	+	4.69E-06
Response to stimulus	62.42	1.51	+	4.46E-03

**GO cellular component complete**	**Expected Value**	**Fold Enrichment**	**+/-**	**P value**
Serine-type endopeptidase complex	0.14	29.31	+	3.09E-02
Serine-type peptidase complex	0.14	29.31	+	3.09E-02
High-density lipoprotein particle	0.27	18.81	+	1.86E-02
Plasma lipoprotein particle	0.33	15.13	+	4.87E-02
Lipoprotein particle	0.33	15.13	+	4.87E-02
Myelin sheath	1.54	7.12	+	1.10E-03

Collagen-containing extracellular matrix	2.84	7.03	+	3.02E-08
Extracellular matrix	3.79	5.28	+	3.66E-06
External encapsulating structure	3.8	5.26	+	3.89E-06
Extracellular space	15.31	4.96	+	2.06E-31
Extracellular region	20.65	4.02	+	9.12E-29
Cytosol	27.91	2.22	+	3.00E-07
Intrinsic component of membrane	44.01	0.27	-	4.77E-07
Integral component of membrane	42.72	0.26	-	5.18E-07

Supplemental Table 3. Enrichment of GO biological processes and GO cellular components from mass spectrometric analysis of serum from repetitive bleomycin mice treated with vehicle or ABT-263.

Name	Sequence (5'-3')	Strand
Bcl2l1	5'-/56-FAM/CACAGAGCA/ZEN/GACCCAGTAAGTGAGC/31ABkFQ/-3'	Probe
(Bcl-xl)	5'-ATTCAGCACGAGCAGTCAG-3'	Primer 1
	5'-CTCAACCAGTCCATTGTCCAA-3'	Primer 2
Bcl2l11	5'-/56-FAM/CCAAGCAAC/ZEN/CTTCTGATGT AAGTTCTGAGT /31ABkFQ/-3'	Probe
(Bim)	5'-TGTCTGACTCTGATTCTCGGA-3'	Primer 1
	5'-TGCAATTGTCCACCTTCTCTG-3'	Primer 2
Bid	5'-/ 56-FAM/ AGTTCCTTT /Z EN/TGTCTTCCTCCGACAGG /31ABkFQ/-3'	Probe
	5'-GCCGCACAGTTCATGAATG-3'	Primer 1
	5'-CCTTGTCGTTCTCCATGTCTC-3'	Primer 2
Bak1	5'-/56-FAM/CCCGTCCCC/ZEN/TTCTGAACAGCA/31ABkFQ/-3'	Probe
	5'-AATGGCATCTGGACAAGGAC-3'	Primer 1
	5'-CTTCGAAAGACCTCCTCTGTG-3'	Primer 2
Bax	5'-/ 56-FAM/CGTCAGCAA/Z EN/TCA TCCTCTGCAGCT /31ABkFQ/-3'	Probe
	5'-GGAGATGAACTGGACAGCAAT-3'	Primer 1
	5'-GCCATCAGCAAACATGTCAG-3'	Primer 2
Mcl1	5'-/ 56-FAM/ AGAGGCTGG /ZEN/GA TGGGTTTGTGG /31ABkFQ/-3'	Probe
	5'-ACAGATGTTCTTGTAAGGACGA-3'	Primer 1
	5'-CTTCTAGGTCCTGTACGTGGA-3'	Primer 2
Bcl2l2	5'-/ 56-FAM/ ACCA TCCAA/Z EN/TCCTGCACTTGTCCC/31ABkFQ/-3'	Probe
(Bcl-w)	5'-TGTGTGCTGAGAGTGTCAAC-3'	Primer 1
	5'-CCGTATAGAGCTGTGAACTCC-3'	Primer 2
Bcl2	5'- /56-FAM/CAG ATT GGG /ZEN/TCC TCA CAC TCC GG/31ABkFQ/ -3'	Probe
	5'-TTG TAA TTC ATC TGC CGC CG-3'	Forward
	5'- AAT GAA TCG GGA GTT GGG GT -3'	Reverse
Acta2	/5TET/CA GCA CAG C/ZEN/C TGA ATA GCC ACA TAC A/3IABkFQ/ -3’	Probe
	5’- GAG TCC AGC ACA ATA CCA GTT -3’	Forward
	5’- CAC TGA ACC CTA AGG CCA AC -3’	Reverse
GusB	5'- /56-FAM/ACCTCCAAA/ZEN/TGCCCATAGTCATGATACC/31ABkfq/ -3'	Probe
	5'- GATGCGTCTTATACCAGTTCTCA -3'	Primer 1
	5'- CAACGCCAAATATGATGCAGAC -3'	Primer 2

Supplemental Table 4. Primer sequences used for qPCR.

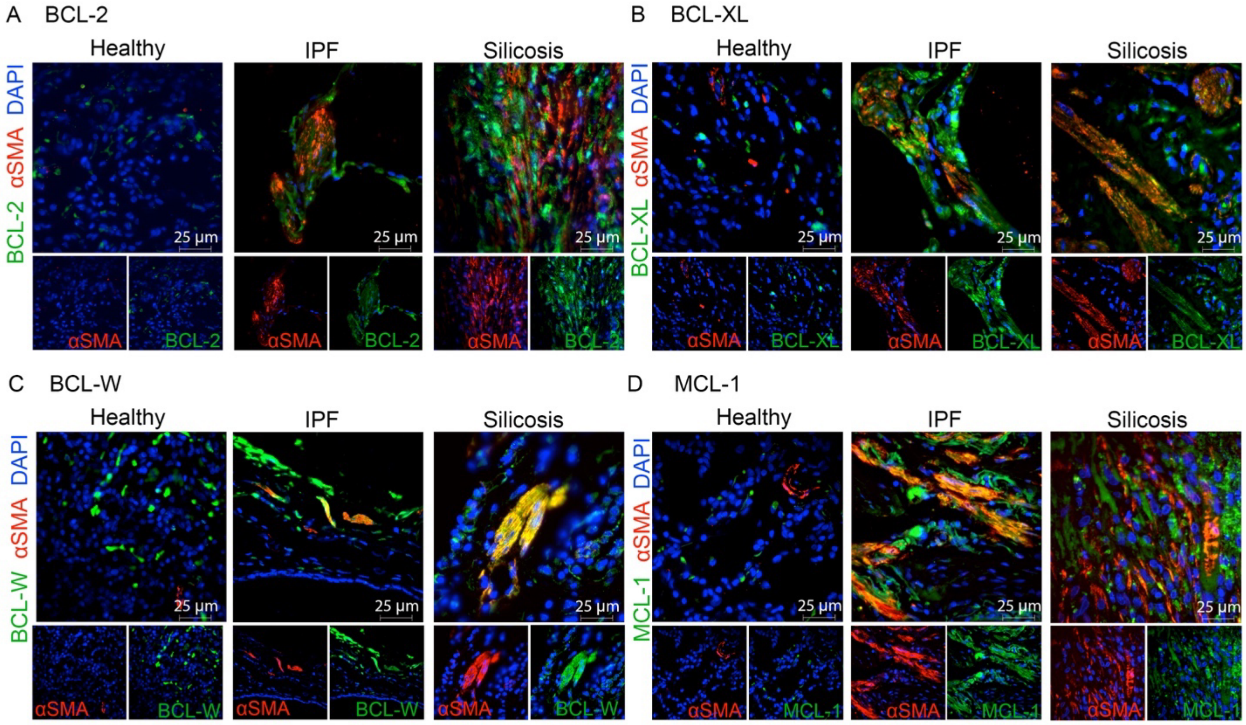

Supplemental Figure 1. αSMA+ fibrotic fibroblasts express anti-apoptotic BCL-2 family members in IPF and silicosis (40X images). Immunofluorescent imaging of lungs from healthy donors, IPF, and silicosis for anti-αSMA (red), DAPI (blue) and anti-apoptotic BCL-2 family members (green) (A) BCL-2, (B) BCL-XL, (C) BCL-W, (D) MCL-1. Total magnification: 400X

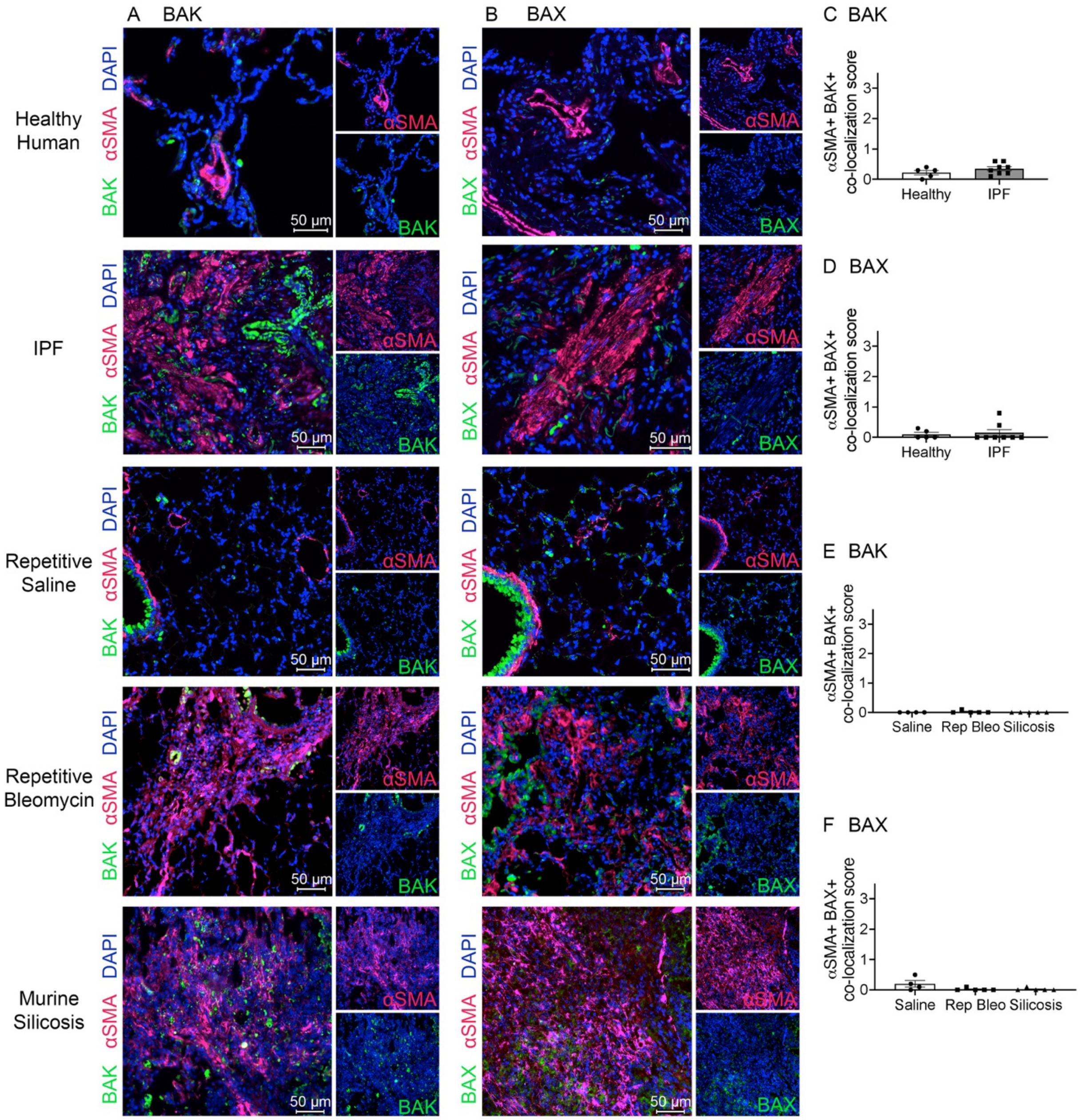

Supplemental Figure 2. αSMA+ fibroblasts do not express significant BAX or BAK in human or murine fibrotic lung disease. Immunofluorescent imaging of lungs from human (healthy donors, IPF) and mice (repetitive saline, 28-week repetitive bleomycin, and 24-week silicosis). Samples were stained for anti-αSMA (red), DAPI (blue) and pro-apoptotic BCL-2 family members (green) (A) BAK and (B) BAX, with scoring of co-localization within αSMA+ cells for BAK in (C) human and (D) mouse and BAX in (E) human and (F) mouse. n=4-8 per group. Semiquantitative scoring of co-localization of BAX or BAK in αSMA+ cells: 0 (0%), 1 (1-33%), 2

(34-66%), 3 (67-100%). n=4-8 samples per group. Ten images per slide were scored. Graphed as scatter plot with bar, mean +/- SEM. *$P<0.05$,* Brown-Forsythe and Welch's ANOVA with Dunnett correction for multiple comparisons. Total magnification 200X.

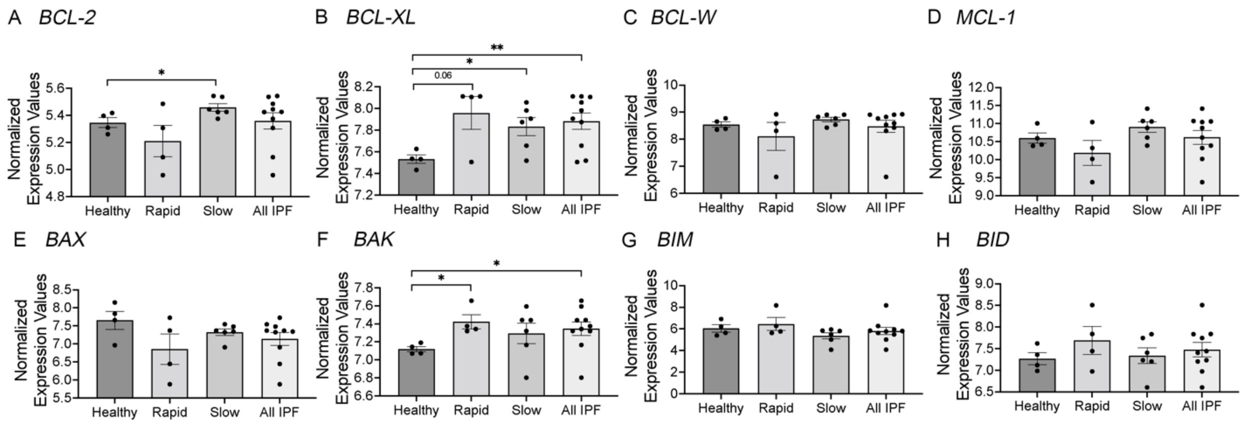

Supplemental Figure 3. IPF fibrotic fibroblasts have increased expression *of BCL-2, BCL-XL*, and *BAK* compared to healthy fibroblasts. RNA expression as determined by microarray analysis of in vitro cultured primary lung fibroblasts from healthy donors and patients with rapidly progressing and slowly progressing IPF for BCL-2 family members: (A) *BCL-2*, (B) *BCL-XL*, (C) *BCL-W*, (D) *MCL-1*, (E) *BAX*, (F) *BAK*, (G) *BIM*, and (H) *BID*. GSE 44723: Healthy n=4, rapid n=4, slow n=6, all IPF n=10. Graphed as scatter plot with bar, mean +/- SEM. **$P<0.05$, **$P<0.01$, ***$P<0.001$*, 2-tailed t-test with Welch's correction.

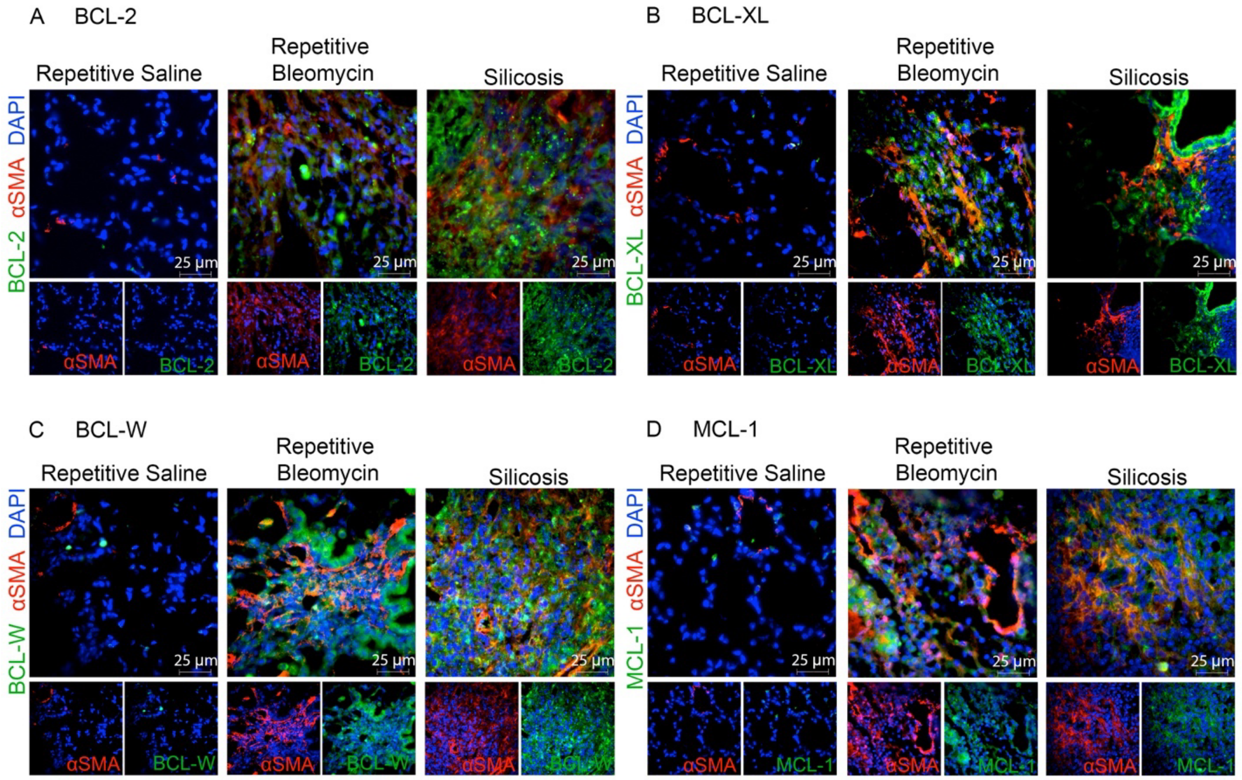

Supplemental Figure 4. In two pre-clinical models of PF-ILD, αSMA+ fibrotic fibroblasts express anti-apoptotic BCL-2 family members (40X). Immunofluorescent imaging of murine lungs from repetitive saline treated, repetitive bleomycin (28-week), and silicosis (24-week) mice for anti-αSMA (red), DAPI (blue) and anti-apoptotic BCL-2 family members (green) (A) BCL-2, (B) BCL-XL, (C) BCL-W, (D) MCL-1. Total magnification 400X.

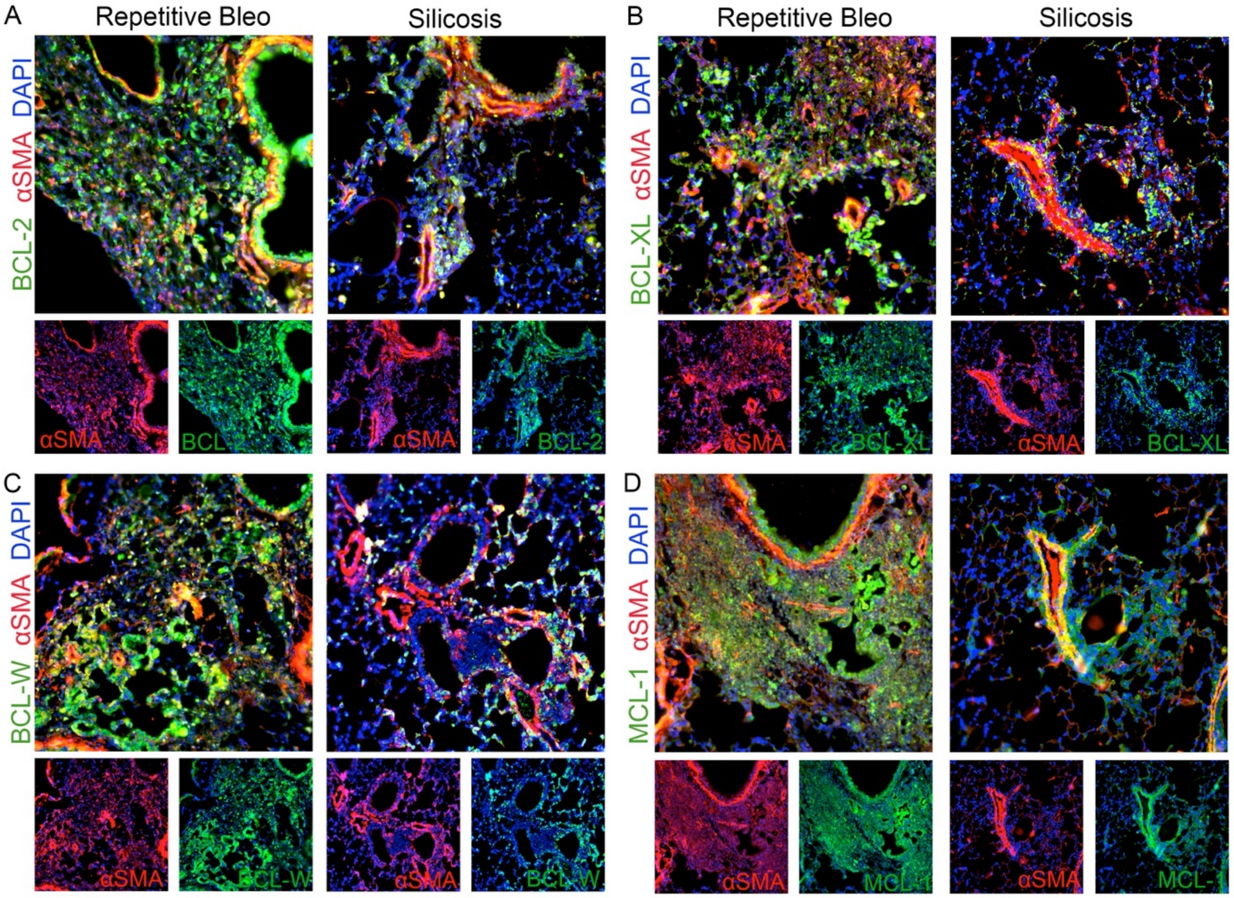

Supplemental Figure 5. In earlier stages of two murine models of PF-ILD, αSMA+ fibrotic fibroblasts express anti-apoptotic BCL-2 family members. Immunofluorescent imaging of murine lungs from naïve, repetitive bleomycin (10-week), and murine silicosis (4-week) for anti-αSMA (red), DAPI (blue) and anti-apoptotic BCL-2 family members (green) (A) BCL-2, (B) BCL-XL, (C) BCL-W, (D) MCL-1. Total magnification 200X.

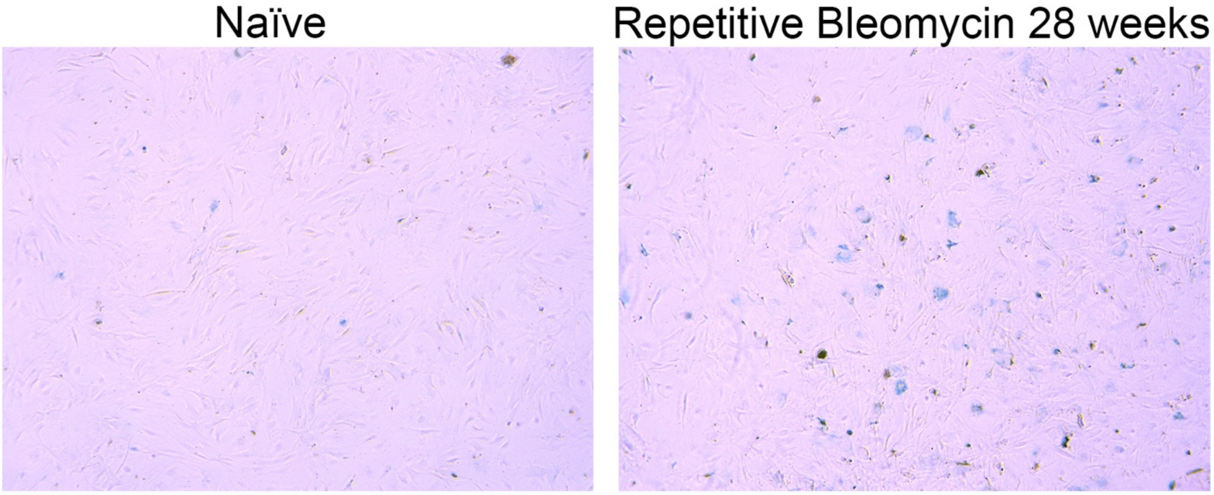

Supplemental Figure 6. Fibroblasts from repetitive bleomycin treated mice have increased markers of senescence. Beta-galactosidase staining (blue) of in vitro cultured primary lung fibroblasts from naive and 28-week repetitive bleomycin mice. Total magnification 50X.

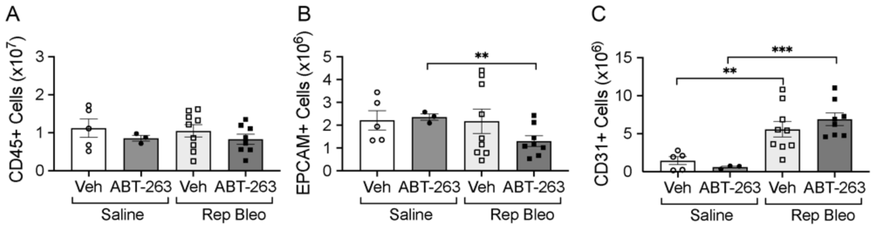

Supplemental Figure 7. After repetitive bleomycin, treatment with ABT-263 did not alter epithelial cell, endothelial cell, or leukocyte numbers. Quantification of (A) leukocyte (CD45+), (B) epithelial cell (EPCAM+), and (C) endothelial cell (CD31+) populations by flow cytometry. n= 3-9 mice per group. Graphed as scatter plot with bar, mean +/- SEM. **P<0.05, **P<0.01, ***P<0.001*, 2-tailed t-test with Welch's correction.

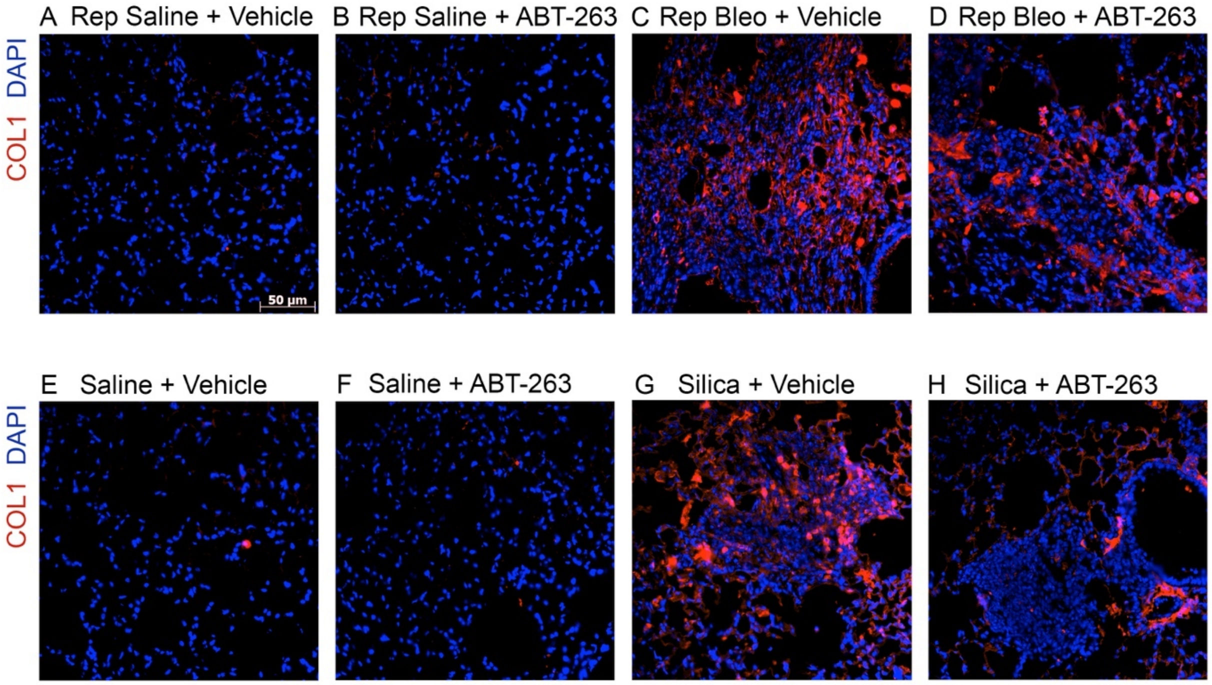

Supplemental Figure 8. After repetitive bleomycin or silica, treatment with ABT-263 reduces COL1 staining. Immunofluorescent imaging of murine lungs stained for DAPI (blue) and COL1 (red) after treatment with (A) repetitive saline + vehicle, (B) repetitive saline + ABT-263, (C) repetitive bleomycin + vehicle, (D) repetitive bleomycin + ABT-263, (E) saline + vehicle, (F) saline + ABT-263, (G) silica + vehicle, (H) silica + ABT-263. Total magnification 200X.

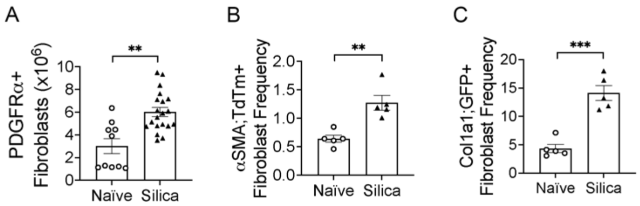

Supplemental Figure 9. Eight-week silica mice have increased PDGFRα+, αSMA+, and Col1a1+ fibrotic fibroblasts. Lin- fibroblasts isolated from αSMA;TdTm and Col1a1;GFP were quantified by flow cytometry for (A) PDGFRα+, (B) αSMA;TdTm+, and (C) Col1a1;GFP+ cells. n=5 per group (αSMA;TdTm and Col1a1;GFP), n=10 per group (PDGFRα). Graphed as scatter plot with bar, mean +/- SEM. **P<0.05, **P<0.01, ***P<0.001*, 2-tailed t-test with Welch's correction.

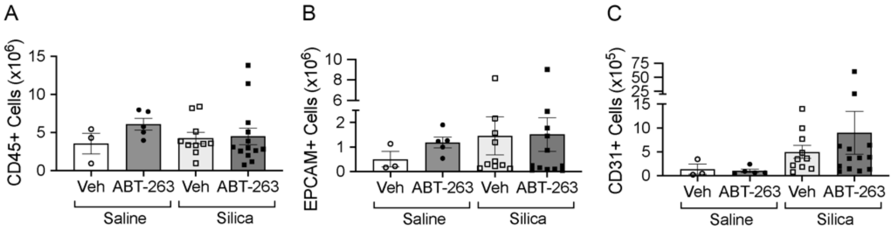

Supplemental Figure 10. After silica exposure, treatment with ABT-263 did not alter epithelial cell, endothelial cell, or leukocyte numbers. Quantification of (A) leukocyte (CD45+), (B) epithelial cell (EPCAM+), and (C) endothelial cell (CD31+) populations by flow cytometry. n=3-13 mice per group. Graphed as scatter plot with bar, mean +/- SEM. **P<0.05, **P<0.01, ***P<0.001,* 2-tailed t-test with Welch's correction.

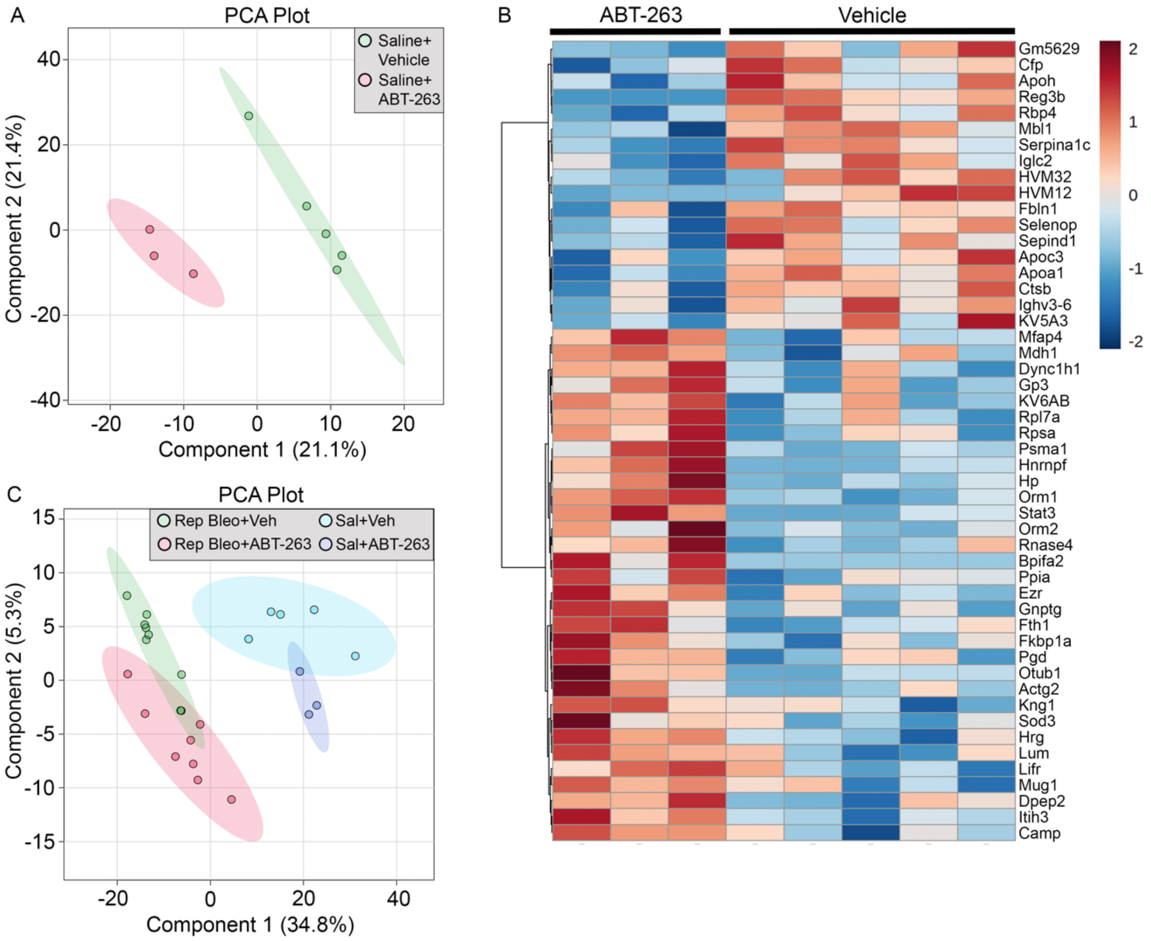

Supplemental Figure 11. Serum proteomic analysis separates vehicle and ABT-263 treated populations. PCA of global proteomic patterns between (A) vehicle and ABT-263 treated repetitive saline instilled mice, and (B) vehicle and ABT-263 treated mice after repetitive saline or repetitive bleomycin. (C) Heat map of normalized signal between repetitive saline mice treated with vehicle and ABT-263. n=3-9 mice per group.

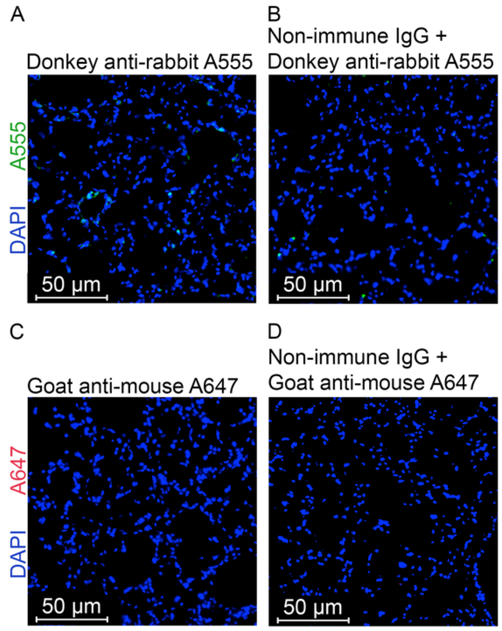

Supplemental Figure 12. Negative controls for immunofluorescent imaging. Immunofluorescent imaging of lungs from naïve mice treated with DAPI (blue) and (A) secondary donkey anti-rabbit A555, (B) non-immune IgG with secondary donkey anti-rabbit A555, (C) secondary goat anti-mouse A647 and (D) non-immune IgG with secondary goat anti-mouse A647. Total magnification 200X.

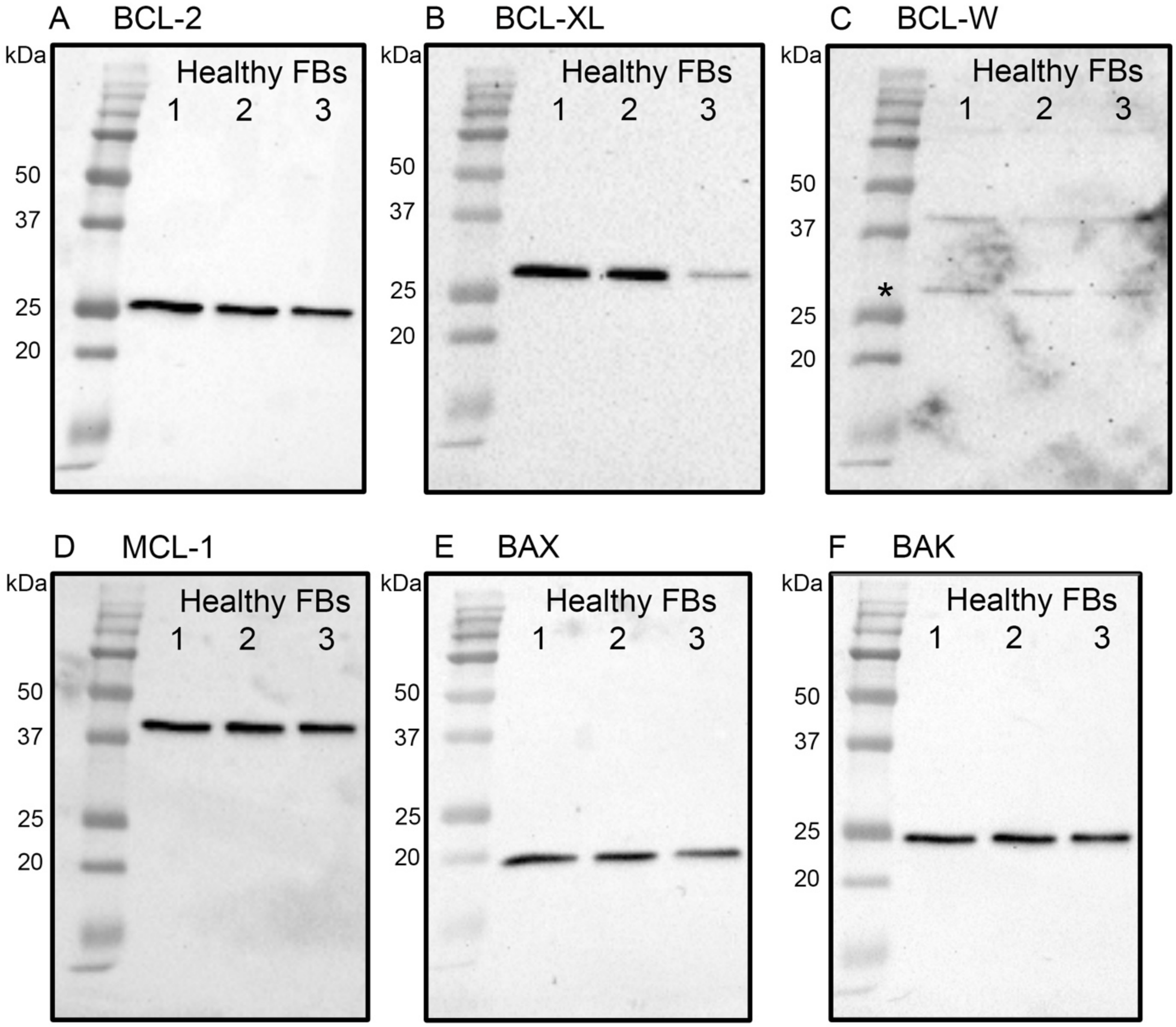

Supplemental Figure 13. Western blots from healthy human fibroblasts grown in vitro for (A) BCL-2, (B) BCL-XL, (C) BCL-W, (D) MCL-1, (E) BAX and (F) BAK.

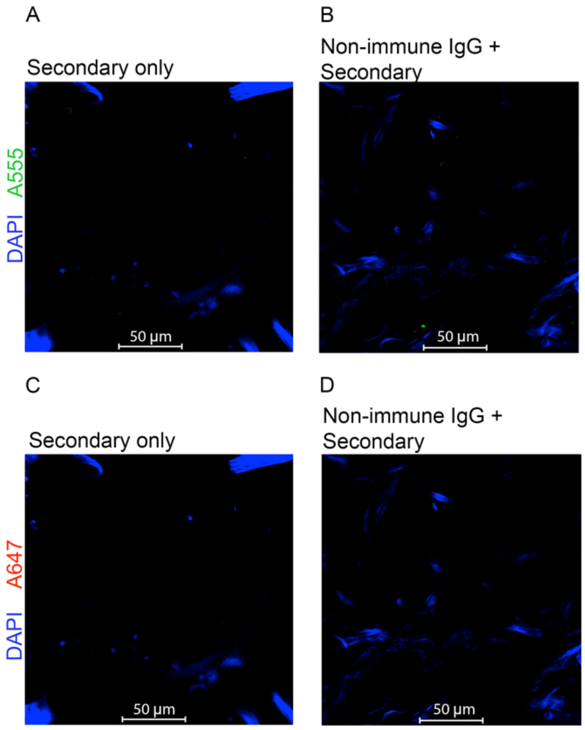

Supplemental Figure 14. Negative controls for precision cut lung slices. Immunofluorescent images of precision cut lung slices from an IPF lung stained with DAPI (blue), secondary donkey anti-rabbit A555 (green), secondary goat anti-mouse A647 without (A,C) and with (B,D) non-immune IgG. Total magnification 200X.
